# Benzofuran-Annulated Naphthalimides Trigger Replication Stress, DNA Damage, and p53-Dependent Cell Cycle Arrest

**DOI:** 10.3390/pharmaceutics18060754

**Published:** 2026-06-20

**Authors:** Zlatina Vlahova, Lazar Lazarov, Maria Petrova, Shazie Yusein-Myashkova, Jordana Todorova, Maria Schröder, Monika Mutovska, Stanimir Stoyanov, Yulian Zagranyarski, Iva Ugrinova

**Affiliations:** 1 Institute of Molecular Biology “Acad. Roumen Tsanev”, Bulgarian Academy of Sciences, Acad. G. Bonchev Str., bl. 21, 1113 Sofia, Bulgaria; vlahova94@gmail.com (Z.V.); lazarutko@gmail.com (L.L.); mhristova84@abv.bg (M.P.); shazi@abv.bg (S.Y.-M.); jordanabg@yahoo.com (J.T.); marias82@abv.bg (M.S.); 2 Faculty of Chemistry and Pharmacy, Sofia University “St. Kliment Ohridski”, 1 J. Baurchier Blvd, 1164 Sofia, Bulgaria; ohmgm@chem.uni-sofia.bg (M.M.); ohss@chem.uni-sofia.bg (S.S.)

**Keywords:** benzofuran, naphthalimide, anticancer agents, replication stress, DNA damage, γH2AX, cell cycle arrest, apoptosis, p53, autophagy-related LC3 response

## Abstract

**Background/Objectives:** DNA-targeting small molecules that induce replication stress represent a promising strategy in anticancer drug development. 1,8-Naphthalimide (NI) derivatives are well-established DNA-intercalating agents, and heterocyclic annulation offers a rational approach to enhancing their potency and tumor selectivity. Here, we report the synthesis and biological evaluation of a novel series of benzofuran-containing naphthalimide derivatives, with particular focus on the lead dinitro-substituted compound **5d**. **Methods:** Cytotoxic activity was assessed using the MTT assay in A549 (p53 wild-type), H1299 (p53-null), and MRC-5 cells. Long-term antiproliferative effects were evaluated by clonogenic survival assay. Cell cycle distribution was analyzed by propidium iodide staining and flow cytometry. Replication stress and DNA damage were quantified by EdU incorporation and γH2AX immunofluorescence, respectively. Apoptosis was assessed by Annexin V/PI staining and caspase-3/7 activation assay. p53 nuclear accumulation and autophagy induction were evaluated by immunofluorescence and Western blot, using LC3 as an autophagic marker. **Results:** All compounds exhibited cytotoxic activity in the nanomolar range, with **5d** emerging as the most potent and selective. Clonogenic survival was significantly reduced, indicating durable suppression of proliferative capacity. Treatment with **5d** induced G_1_ arrest in A549 cells and the accumulation of H1299 cells in G_2_/M, consistent with p53-dependent and p53-independent checkpoint activation, respectively. EdU incorporation was markedly reduced, while γH2AX intensity increased, collectively supporting a replication stress-driven mechanism of DNA damage. Apoptosis was confirmed by increased Annexin V-positive populations and caspase-3/7 activation. LC3 puncta formation and LC3-I/LC3-II conversion were increased, indicating LC3 processing and autophagosome accumulation consistent with the activation of autophagy-related processes. **Conclusions: 5d** induces a cellular phenotype consistent with replication stress, including reduced EdU incorporation, γH2AX accumulation, cell cycle arrest, and apoptotic cell death in a p53 status-dependent manner. These findings establish benzofuran-annulated naphthalimides as a promising scaffold for the development of anticancer agents that exploit replication stress vulnerabilities in tumor cells.

## 1. Introduction

Cancer remains a leading cause of death worldwide, driven by the accumulation of genetic and epigenetic alterations that promote uncontrolled proliferation, resistance to cell death, and genomic instability [1,2]. Among the numerous therapeutic strategies, targeting DNA integrity and replication dynamics is still a cornerstone of anticancer drug development, particularly through agents that interfere with DNA topology and replication stress responses [3,4,5].

DNA-interacting small molecules, including classical intercalators such as anthracyclines and naphthalimides, exert their cytotoxic effects by perturbing DNA structure and stabilizing transient DNA–enzyme cleavage complexes, most notably those involving topoisomerases [6,7]. These interactions result in replication fork stalling, accumulation of DNA double-strand breaks, and activation of the DNA damage response (DDR), ultimately leading to cell cycle arrest and apoptosis [8,9,10,11]. The cellular response to such stress is highly dependent on the integrity of checkpoint pathways, particularly the p53 axis, which governs cell-fate decisions following genotoxic insult.

In this context, 1,8-naphthalimide (NI) derivatives have emerged as a versatile class of DNA-targeting agents with well-documented anticancer activity, exemplified by clinically investigated compounds such as mitonafide and amonafide [12,13,14,15]. Their planar aromatic structure enables efficient DNA intercalation, while chemical modifications allow fine-tuning of binding affinity, selectivity, and cellular responses [16,17]. Despite these advances, dose-limiting toxicity, acquired resistance, and insufficient tumor selectivity continue to drive the search for novel derivatives with improved pharmacological profiles.

One promising strategy involves extending the aromatic system through fusion with additional heterocycles, thereby enhancing π–π stacking interactions and modulating electronic properties. Among such scaffolds, benzofuran represents a privileged structural motif in medicinal chemistry, associated with a broad spectrum of biological activities, including anticancer, anti-inflammatory, and antimicrobial effects [18,19,20]. The rigid, conjugated nature of benzofuran facilitates strong interactions with nucleic acids and protein targets, while the incorporation of electron-withdrawing substituents, such as nitro groups, can further enhance biological activity by modulating redox properties and electrophilicity [21,22].

Recent studies have shown that benzofuran-containing compounds can act as topoisomerase inhibitors and replication stress inducers, leading to the activation of ATR/CHK1 signaling and accumulation of DNA damage markers such as γH2AX [23,24,25]. These mechanisms are particularly relevant in the context of targeting rapidly proliferating tumor cells characterized by elevated baseline replication stress, a hallmark of oncogenic transformation [26,27].

Building on these concepts, the design of hybrid molecules that combine the DNA-intercalating naphthalimide core with benzofuran moieties represents a rational approach to enhancing anticancer activity through synergistic structural and electronic effects. Such hybrids are expected to exhibit increased planarity, extended conjugation, and improved DNA-binding properties, potentially translating into greater potency in inducing replication stress and cytotoxicity.

In the present study, we report the synthesis and biological evaluation of a novel series of benzofuran-containing naphthalimide derivatives. We hypothesized that the extension of the naphthalimide aromatic system through benzofuran annulation, combined with strategic nitro group substitution, would enhance DNA-targeting potency and induce replication stress in cancer cells in a p53 status-dependent manner. To test this hypothesis, we assessed cytotoxic activity in human cancer cell lines with a distinct p53 status, and explored their mechanism of action in the context of DNA damage induction, replication stress, and apoptosis. Our findings provide new insights into the structure–activity relationships of these hybrid molecules and support their potential as next-generation DNA-targeting anticancer agents.

## 2. Materials and Methods

### 2.1. Synthesis

All starting materials and solvents were commercially available and used without further purification. Reagents were purchased from Fluorochem (Glossop, UK), Acros Organics (Antwerp, Belgium), and Fisher Scientific (Hampton, NH, USA). NMR spectra were recorded on a Bruker Avance 500 MHz spectrometer (Bruker, Karlsruhe, Germany) operating at 500 and 126 MHz for ^1^H and ^13^C NMR, respectively. Chloroform-*d* and trifluoroacetic acid-*d* were used as solvents. Chemical shifts (δ) are reported in ppm and referenced to residual solvent signals (^1^H: 7.26 ppm and ^13^C: 77.16 ppm for Chloroform-*d*; ^1^H: 11.50 ppm and ^13^C: 164.20 ppm for trifluoroacetic acid-*d*). High-resolution mass spectrometry (HRMS) data were recorded on a Thermo Fisher Scientific Orbitrap Exploris 120 instrument (Bremen, Germany) equipped with a combined HESI/APCI source. Elemental analyses were carried out using a Leco CHNS-932 analyzer (Leco Europe, Geleen, The Netherlands). Thin-layer chromatography (TLC) was performed on silica gel plates (Macherey–Nagel F60 254, 40 × 80 mm, 0.2 mm thickness; Düren, Germany).

**General procedure for the preparation of esters 2a–d:** A mixture of dibutyl 3,4-dibromo-6-nitronaphthalene-1,8-dicarboxylate (10.0 mmol, 5.31 g), the corresponding phenol (12.0 mmol), and potassium carbonate (12.0 mmol, 1.66 g) in NMP (40 mL) was stirred at 150 °C for 20–40 min under air. After cooling to room temperature, the reaction mixture was poured into a mixture of ice and concentrated hydrochloric acid (10 mL). For compounds **2a** and **2b**, which were obtained as oils, the aqueous phase was extracted with dichloromethane. The combined organic layers were washed with water, dried over anhydrous sodium sulfate, filtered, and concentrated under reduced pressure. For the remaining compounds, the resulting precipitate was filtered, washed with water, and dried under reduced pressure. The crude products were purified by column chromatography on silica gel using a gradient of cyclohexane to cyclohexane/dichloromethane (1:1) as eluent.

#### 2.1.1. Dibutyl 3-Bromo-6-nitro-4-phenoxynaphthalene-1,8-dicarboxylate **2a**

Yield 5.06 g (93%) as brownish oil. ^1^H NMR (Chloroform-*d*, 500 MHz): δ 9.06 (d, J = 2.4 Hz, 1H), 8.72 (d, J = 2.4 Hz, 1H), 8.36 (s, 1H), 7.36–7.29 (m, 2H), 7.11 (t, J = 7.4 Hz, 1H), 6.86–6.81 (m, 2H), 4.38 (t, J = 6.8 Hz, 2H), 4.36 (t, J = 6.8 Hz, 2H), 1.80 (h, J = 6.9 Hz, 4H), 1.53–1.46 (m, 4H), 1.01 (t, J = 7.4 Hz, 3H), 1.00 (t, J = 7.4 Hz, 3H). ^13^C{^1^H} NMR (Chloroform-*d*, 126 MHz): 166.85, 166.60, 157.43, 152.11, 145.62, 137.94, 133.42, 130.69, 130.33, 130.26, 129.07, 127.86, 123.67, 123.54, 122.38, 115.67, 66.44, 66.29, 30.68, 30.66, 19.33, 13.89. Anal. calcd. C_26_H_26_BrNO_7_ C, 57.36; H, 4.81; N, 2.57%; found C, 57.23; H, 4.89; N, 2.43%.

#### 2.1.2. Dibutyl 3-Bromo-6-nitro-4-(2-nitrophenoxy)naphthalene-1,8-dicarboxylate **2b**

Yield 5.07 g (86%).as brownish oil. ^1^H NMR (Chloroform-*d*, 500 MHz): 9.17 (d, J = 2.3 Hz, 1H), 8.76 (d, J = 2.4 Hz, 1H), 8.35 (s, 1H), 8.11 (dd, J = 8.2, 1.7 Hz, 1H), 7.41 (ddd, J = 8.7, 7.4, 1.7 Hz, 1H), 7.25 (ddd, J = 8.5, 7.4, 1.2 Hz, 1H), 6.45 (dd, J = 8.4, 1.1 Hz, 1H), 4.38 (t, J = 6.8 Hz, 2H), 4.36 (t, J = 6.8 Hz, 2H), 1.84–1.76 (m, 4H), 1.54–1.45 (m, 4H), 1.01 (d, J = 7.4 Hz, 3H), 1.00 (t, J = 7.4 Hz, 3H). ^13^C{1H} NMR (Chloroform-*d*, 126 MHz): 166.66, 166.34, 150.69, 149.81, 146.02, 139.78, 137.58, 134.54, 133.61, 130.60, 130.07, 129.78, 126.74, 124.00, 123.73, 121.88, 116.25, 115.11, 66.56, 66.45, 30.65, 19.33, 13.88. Anal. calcd. C_26_H_25_BrN_2_O_9_ C, 52.98; H, 4.28; N, 4.75%; found C, 53.11; H, 4.35; N, 4.90%.

#### 2.1.3. Dibutyl 3-Bromo-6-nitro-4-(3-nitrophenoxy)naphthalene-1,8-dicarboxylate **2c**

Yield 5.89 g (100%) as brownish oil. ^1^H NMR (Chloroform-*d*, 500 MHz): 9.00 (d, J = 2.4 Hz, 1H), 8.76 (d, J = 2.4 Hz, 1H), 8.38 (s, 1H), 8.01 (ddd, J = 8.2, 2.0, 0.8 Hz, 1H), 7.64 (t, J = 2.3 Hz, 1H), 7.55 (t, J = 8.3 Hz, 1H), 7.24 (ddd, J = 8.3, 2.6, 0.8 Hz, 1H), 4.39 (t, J = 6.8 Hz, 2H), 4.37 (t, J = 6.8 Hz, 2H), 1.85–1.77 (m, 4H), 1.54–1.46 (m, 4H), 1.01 (t, J = 7.4 Hz, 3H), 1.01 (t, J = 7.4 Hz, 3H). ^13^C{^1^H} NMR (Chloroform-*d*, 126 MHz): 166.62, 166.32, 157.40, 150.75, 149.63, 145.90, 137.77, 133.79, 131.11, 130.69, 130.07, 129.79, 123.95, 122.00, 121.54, 118.68, 115.32, 110.81, 66.57, 66.46, 30.65, 19.33, 13.88. Anal. calcd. C_26_H_25_BrN_2_O_9_ C, 52.98; H, 4.28; N, 4.75%; found C, 53.09; H, 4.22; N, 4.85%.

#### 2.1.4. Dibutyl 3-Bromo-6-nitro-4-(4-nitrophenoxy)naphthalene-1,8-dicarboxylate **2d**

Yield 5.72 g (97%) as white crystals. ^1^H NMR (Chloroform-*d*, 500 MHz): 8.96 (d, J = 2.4 Hz, 1H), 8.76 (d, J = 2.3 Hz, 1H), 8.37 (s, 1H), 8.26 (d, J = 9.2 Hz, 2H), 6.96 (d, J = 9.2 Hz, 2H), 4.39 (t, J = 6.8 Hz, 2H), 4.37 (t, J = 6.8 Hz, 2H), 1.81 (h, J = 7.0 Hz, 4H), 1.53–1.45 (m, 4H), 1.01 (t, J = 7.4 Hz, 3H), 1.00 (t, J = 7.4 Hz, 3H). ^13^C{1H} NMR (Chloroform-*d*, 126 MHz): 166.62, 166.31, 161.42, 150.65, 145.93, 143.79, 137.62, 133.82, 130.65, 130.20, 129.61, 126.58, 123.93, 121.43, 116.09, 115.36, 66.61, 66.50, 30.65, 19.32, 13.88. Anal. calcd C_26_H_25_BrN_2_O_9_ C, 52.98; H, 4.28; N, 4.75%; found C, 52.90; H, 4.37; N, 4.77%.

**General procedure for the preparation of esters 3a–d:** A mixture of the corresponding 3-bromodibutyl ester (10.0 mmol), potassium carbonate (30 mmol, 4.15 g, 3.0 equiv), palladium acetate (0.30 mmol, 0.067 g, 3 mol %), triphenylphosphine (0.75 mmol, 0.197 g, 7.5 mol %), pivalic acid (3.0 mmol, 0.31 g, 30 mol %), and 18-crown-6 (0.05 g) in xylene (40 mL) was heated at 130 °C under argon until complete consumption of the starting ester, as monitored by TLC. The reaction mixture was cooled to room temperature and extracted with dichloromethane. The organic layer was dried, filtered, and concentrated under reduced pressure. The crude products were purified by column chromatography on silica gel using a gradient of cyclohexane to cyclohexane/dichloromethane (1:1) as eluent.

#### 2.1.5. Dibutyl 2-Nitronaphtho[1,2-b]benzofuran-4,5-dicarboxylate **3a**

Yield 4.45 g (96%) as white crystals. ^1^H NMR (Chloroform-*d*, 500 MHz): 9.48 (d, J = 2.3 Hz, 1H), 8.73 (d, J = 2.4 Hz, 1H), 8.72 (s, 1H), 8.05 (d, J = 7.7 Hz, 1H), 7.77 (d, J = 8.3 Hz, 1H), 7.60 (td, J = 8.4, 7.9, 1.2 Hz, 2H), 7.49 (d, J = 7.5 Hz, 1H), 4.40 (t, J = 6.8 Hz, 2H), 4.39 (t, J = 6.8 Hz, 2H), 1.82 (*p*, J = 7.0 Hz, 4H), 1.51 (h, J = 7.4 Hz, 4H), 1.01 (td, J = 7.4, 1.3 Hz, 6H). ^13^C{^1^H} NMR (Chloroform-*d*, 126 MHz): 168.11, 167.33, 156.79, 154.39, 144.86, 133.58, 129.52, 128.40, 127.18, 126.13, 124.42, 123.64, 122.70, 121.65, 121.17, 120.77, 120.40, 112.51, 66.25, 65.92, 30.78, 30.69, 19.40, 19.36, 13.94, 13.91. Anal. Calcd for C_26_H_25_NO_7_: C, 67.38; H, 5.44; N, 3.02. Found: C, 67.24; H, 5.59; N, 3.19.

#### 2.1.6. Dibutyl 2,10-Dinitronaphtho[1,2-b]benzofuran-4,5-dicarboxylate **3b**

Yield 4.58 g (90%) as pale-yellow crystals. ^1^H NMR (Chloroform-*d*, 500 MHz): 9.61 (d, J = 2.4 Hz, 1H), 8.83 (d, J = 2.4 Hz, 1H), 8.75 (s, 1H), 8.48–8.43 (m, 1H), 8.42–8.38 (m, 1H), 7.67 (t, J = 7.9 Hz, 1H), 4.40 (q, J = 6.7 Hz, 4H), 1.87–1.76 (m, 4H), 1.51 (ddd, J = 14.9, 7.4, 5.7 Hz, 4H), 1.01 (t, J = 7.4 Hz, 3H), 1.01 (t, J = 7.4 Hz, 3H). ^13^C{^1^H} NMR (Chloroform-*d*, 126 MHz): 167.66, 167.03, 155.26, 148.83, 145.44, 134.79, 133.81, 130.30, 127.87, 127.67, 127.46, 126.35, 124.57, 124.23, 123.69, 121.72, 120.87, 118.94, 66.46, 66.19, 30.74, 30.68, 19.38, 19.35, 13.92, 13.90. Anal. Calcd for C_26_H_24_N_2_O_9_: C, 61.42; H, 4.76; N, 5.51. Found: C, 61.60; H, 4.71; N, 5.61.

#### 2.1.7. Dibutyl 2,9-Dinitronaphtho[1,2-b]benzofuran-4,5-dicarboxylate **3c**

Yield 4.53 g (89%) pale-yellow crystals. ^1^H NMR (Chloroform-*d*, 500 MHz): 9.53 (d, J = 2.4 Hz, 1H), 8.82 (d, J = 2.4 Hz, 1H), 8.77 (s, 1H), 8.69 (d, J = 1.9 Hz, 1H), 8.45 (dd, J = 8.6, 2.0 Hz, 1H), 8.21 (d, J = 8.6 Hz, 1H), 4.40 (t, J = 6.8 Hz, 2H), 4.39 (t, J = 6.9 Hz, 2H), 1.85–1.79 (m, 4H), 1.55–1.47 (m, 4H), 1.01 (t, J = 7.4 Hz, 6H). ^13^C{^1^H} NMR (Chloroform-*d*, 126 MHz): 167.59, 166.95, 156.95, 155.58, 147.56, 145.35, 133.99, 130.63, 129.38, 127.63, 126.87, 123.86, 121.70, 121.35, 120.84, 120.09, 118.91, 108.98, 66.49, 66.20, 30.74, 30.67, 19.38, 19.34, 13.92, 13.90. Anal. Calcd for C_26_H_24_N_2_O_9_: C, 61.42; H, 4.76; N, 5.51. Found: C, 61.49; H, 4.81; N, 5.72.

#### 2.1.8. Dibutyl 2,8-dinitronaphtho[1,2-b]benzofuran-4,5-dicarboxylate **3d**

Yield 4.68 g (92%) as pale-yellow crystals. ^1^H NMR (Chloroform-*d*, 500 MHz): 9.48 (d, J = 2.3 Hz, 1H), 8.99 (d, J = 2.1 Hz, 1H), 8.77 (d, J = 2.3 Hz, 1H), 8.75 (s, 1H), 8.54 (dd, J = 9.0, 2.2 Hz, 1H), 7.91 (d, J = 9.0 Hz, 1H), 4.41 (t, J = 6.8 Hz, 2H), 4.40 (t, J = 6.8 Hz, 2H), 1.84 (h, J = 7.1 Hz, 4H), 1.51 (dq, J = 15.1, 7.6 Hz, 4H), 1.03 (t, J = 7.5 Hz, 3H), 1.01 (t, J = 7.5 Hz, 3H). ^13^C{^1^H} NMR (Chloroform-*d*, 126 MHz): 167.56, 166.97, 159.28, 155.88, 145.28, 145.11, 134.00, 130.34, 127.62, 126.52, 124.51, 123.95, 123.49, 121.62, 120.47, 119.57, 117.71, 113.08, 66.48, 66.23, 30.77, 30.66, 19.40, 19.34, 13.93, 13.90. Anal. Calcd for C_26_H_24_N_2_O_9_: C, 61.42; H, 4.76; N, 5.51. Found: C, 61.49; H, 4.52; N, 5.64.

**General procedure for the preparation of anhydrides 4a–d:** To a solution of diesters 3a–d (6.0 mmol) in acetic acid (40 mL), concentrated sulfuric acid (10 mL) was added dropwise under air. The reaction mixture was stirred vigorously and heated at 110 °C for 1 h. After cooling to room temperature, crushed ice (30 g) was added, and the resulting solid was filtered, washed thoroughly with water, and dried under reduced pressure.

#### 2.1.9. 2-Nitro-4H,6H-benzo[de]benzofuro[2,3-g]isochromene-4,6-dione **4a**

Yield 1.86 g (93%) as light-brown crystals. Anal. Calcd for C_18_H_7_NO_6_: C, 64.87; H, 2.12; N, 4.20. Found: C, 64.99; H, 2.23; N, 4.29.

#### 2.1.10. 2,11-Dinitro-4H,6H-benzo[de]benzofuro[2,3-g]isochromene-4,6-dione **4b**

Yield 2.11 g (93%) as light-brown crystals. Anal. Calcd for C_18_H_6_N_2_O_8_: C, 57.16; H, 1.60; N, 7.41. Found: C, 57.02; H, 1.78; N, 7.25.

#### 2.1.11. 2,10-Dinitro-4H,6H-benzo[de]benzofuro[2,3-g]isochromene-4,6-dione **4c**

Yield 2.18 g (96%) as light-brown crystals. Anal. Calcd for C_18_H_6_N_2_O_8_: C, 57.16; H, 1.60; N, 7.41. Found: C, 56.99; H, 1.68; N, 7.44.

#### 2.1.12. 2,9-Dinitro-4H,6H-benzo[de]benzofuro[2,3-g]isochromene-4,6-dione **4d**

Yield 2.25 g (99%) as light-brown crystals. Anal. Calcd for C_18_H_6_N_2_O_8_: C, 57.16; H, 1.60; N, 7.41. Found: C, 57.13; H, 1.55; N, 7.55.

**General procedure for the preparation of imides 5a–d:** To a suspension of the corresponding anhydride **4a**–**d** (5.0 mmol) in 2-methyl-2-butanol (20 mL), *N*,*N*-dimethylethylenediamine (7.5 mmol, 0.66 g) was added under air. The reaction mixture was heated at reflux for 30 min. After cooling to room temperature, crushed ice (20 g) was added, and the resulting solid was filtered, washed thoroughly with water, and dried under reduced pressure.

#### 2.1.13. 5-(2-(Dimethylamino)ethyl)-2-nitro-4H-benzo[de]benzofuro[2,3-g]isoquinoline-4,6(5H)-dione **5a**

Yield 1.92 g (95%) as white crystals. ^1^H NMR (Trifluoroacetic acid-*d*, 500 MHz): 9.65 (s, 1H), 9.48 (s, 1H), 9.40 (d, J = 1.9 Hz, 1H), 8.19 (d, J = 7.7 Hz, 1H), 7.78 (d, J = 8.3 Hz, 1H), 7.71 (t, J = 7.8 Hz, 1H), 7.60 (t, J = 7.5 Hz, 1H), 4.87 (t, J = 5.0 Hz 2H), 3.86 (t, J = 5.1 Hz 2H), 3.32 (s, 6H). ^13^C{^1^H} NMR (Trifluoroacetic acid-*d*, 126 MHz): 168.31, 167.55, 159.65, 159.26, 148.20, 133.21, 132.02, 131.61, 127.58, 126.98, 126.79, 126.17, 125.47, 124.46, 123.22, 121.20, 118.04, 114.44, 60.51, 46.30, 38.75. Anal. Calcd for C_22_H_17_N_3_O_5_: C, 65.50; H, 4.25; N, 10.42. Found: C, 65.38; H, 4.33; N, 10.39. HRMS (ESI) m/z 404.1223 (calcd for C_22_H_18_N_3_O_5_ [M+H]^+^ 404.1241).

#### 2.1.14. 5-(2-(Dimethylamino)ethyl)-2,11-dinitro-4H-benzo[de]benzofuro[2,3-g]isoquinoline-4,6(5H)-dione **5b**

Yield 2.22 g (99%) as brownish crystals. ^1^H NMR (Trifluoroacetic acid-*d*, 500 MHz): 9.72 (d, J = 2.2 Hz, 1H), 9.66 (s, 1H), 9.48 (d, J = 2.2 Hz, 1H), 8.69 (dd, J = 7.8, 1.1 Hz, 1H), 8.62 (d, J = 8.1, 1.1 Hz, 1H), 7.86 (t, J = 8.0 Hz, 1H), 4.93 (t, J = 5.3 Hz 2H), 3.93 (t, J = 5.4 Hz 2H), 3.37 (s, 6H). ^13^C{^1^H} NMR (Trifluoroacetic acid-*d*, 126 MHz): 167.73, 167.17, 159.48, 151.32, 148.84, 136.18, 132.76, 132.48, 131.08, 129.02, 127.87, 127.83, 127.56, 126.49, 126.04, 124.18, 121.27, 120.36, 60.49, 46.33, 38.80. Anal. Calcd for C_22_H_16_N_4_O_7_: C, 58.93; H, 3.60; N, 12.50. Found: C, 59.10; H, 3.67; N, 12.68. HRMS (ESI) m/z 449.1074 (calcd for C_22_H_17_N_4_O_7_ [M+H]^+^ 449.1092).

#### 2.1.15. 5-(2-(Dimethylamino)ethyl)-2,10-dinitro-4H-benzo[de]benzofuro[2,3-g]isoquinoline-4,6(5H)-dione **5c**

Yield 2.09 g (93%) as brownish crystals. ^1^H NMR (Trifluoroacetic acid-*d*, 500 MHz): 10.04 (d, J = 1.9 Hz, 1H), 9.78 (s, 1H), 9.69 (d, J = 2.0 Hz, 1H), 8.95 (d, J = 1.6 Hz, 1H), 8.71 (dd, J = 8.6, 1.5 Hz, 1H), 8.60 (d, J = 8.6 Hz, 1H), 4.98 (t, J = 5.4 Hz 2H), 3.95 (t, J = 5.4 Hz 2H), 3.40 (s, 6H). ^13^C{^1^H} NMR (Trifluoroacetic acid-*d*, 126 MHz): 167.82, 167.22, 161.70, 158.40, 149.91, 148.91, 133.32, 131.46, 128.25, 126.98, 125.92, 124.11, 124.09, 122.82, 121.52, 119.77, 118.13, 117.30, 115.05, 111.10, 60.22, 46.15, 38.62. Anal. Calcd for C_22_H_16_N_4_O_7_: C, 58.93; H, 3.60; N, 12.50. Found: C, 59.02; H, 3.48; N, 12.38. HRMS (ESI) m/z 449.1078 (calcd for C_22_H_17_N_4_O_7_ [M+H]^+^ 449.1092).

#### 2.1.16. 5-(2-(Dimethylamino)ethyl)-2,9-dinitro-4H-benzo[de]benzofuro[2,3-g]isoquinoline-4,6(5H)-dione **5d**

Yield 2.17 g (97%) as brownish crystals. ^1^H NMR (Trifluoroacetic acid-*d*, 500 MHz): 9.90 (d, J = 2.2 Hz, 1H), 9.68 (s, 1H), 9.58 (d, J = 2.1 Hz, 1H), 9.26 (d, J = 2.2 Hz, 1H), 8.66 (dd, J = 9.1, 2.3 Hz, 1H), 8.07 (d, J = 9.1 Hz, 1H), 4.90 (t, J = 5.3 Hz, 2H), 3.88 (t, J = 5.2 Hz, 2H), 3.33 (s, 6H). ^13^C{^1^H} NMR (Trifluoroacetic acid-*d*, 126 MHz): 167.82, 167.32, 162.64, 160.83, 149.01, 147.29, 132.92, 132.66, 128.13, 127.15, 126.79, 126.24, 126.06, 124.85, 121.59, 120.40, 119.98, 60.40, 46.32, 38.79. Anal. Calcd for C_22_H_16_N_4_O_7_: C, 58.93; H, 3.60; N, 12.50. Found: C, 59.03; H, 3.43; N, 12.73. HRMS (ESI) m/z 449.1075 (calcd for C_22_H_17_N_4_O_7_ [M+H]^+^ 449.1092).

### 2.2. Biology

#### 2.2.1. Cell Culture and Compounds

Cell culture conditions and compound preparation were performed as previously described by Vlahova et al. [28]. A549 cells were cultured in DMEM (Thermo Fisher Scientific, Waltham, MA, USA), H1299 cells in RPMI-1640 (Thermo Fisher Scientific, Waltham, MA, USA), and MRC-5 cells in MEM (Thermo Fisher Scientific, Waltham, MA, USA), all supplemented with 10% fetal bovine serum (FBS; Thermo Fisher Scientific, Waltham, MA, USA) and 1% antibiotic–antimycotic solution (Thermo Fisher Scientific, Waltham, MA, USA). Cells were cultured at 37 °C in a humidified atmosphere containing 5% CO_2_. All experiments were initiated when cells reached 70–80% confluence, unless otherwise stated.

Stock solutions of doxorubicin (hydrochloride) (Cayman Chemical Company, Ann Arbor, MI, USA), mitonafide (MedChemExpress LLC, Monmouth Junction, NJ, USA), and the synthesized naphthalimide derivatives were prepared in DMSO at 2 mM and diluted in culture medium to the desired working concentrations prior to use.

#### 2.2.2. Cytotoxicity Test

The cytotoxic activity of the compounds was determined using the MTT-dye reduction assay [29], as described by Vlahova et al. [28]. Cells were plated at an appropriate density for each cell type into 96-well plates (H1299: 1.5 × 10^3^ cells/well; A549: 2.5 × 10^3^ cells/well; MRC-5: 4.5 × 10^3^ cells/well), incubated overnight, and then treated with serially diluted reagents. Four control wells of medium alone provided the blanks for absorbance readings. The concentration of the solvent DMSO in the medium never exceeded 0.5%. After 72 h of treatment, cells were incubated with 0.5 mg/mL MTT solution (Thermo Fisher Scientific, Waltham, MA, USA) for 4 h, and formazan crystals were then solubilized in 100 µL DMSO/well on a shaker for 15 min in the dark. Absorbance was measured using an ELISA plate reader Varioscan (Thermo Fisher Scientific, Waltham, MA, USA) at 570 nm. IC_50_ values were calculated using GraphPad Prism 8.0.1 for Windows (GraphPad Software, Boston, MA, USA).

#### 2.2.3. Colony Formation Assay

Cells were seeded at low densities in 6-well plates (H1299: 600 cells/well; A549: 800 cells/well). After overnight incubation, cells were treated with the calculated IC_10_, IC_15_, and IC_20_ concentrations of the tested compounds for 24 h. After treatment, plates were left in an incubator for 10–14 days, until control cells had formed sufficiently large clones (>50 cells), with medium replaced every 3 days. Colonies were fixed with 3.7% paraformaldehyde, stained with 0.2% crystal violet, and counted using Fiji (ImageJ Software 2.15.1; https://hpc.nih.gov/apps/Fiji.html, accessed on 15 June 2026) [30]. Plating efficiency (PE) and survival fraction (SF) were calculated as described [31]. Dose–survival curves were made using R (version 4.3.2; R Foundation for Statistical Computing, Vienna, Austria), with RStudio (version 2026.04.0; Posit Software, Boston, MA, USA) used as the interface.

#### 2.2.4. Flow Cytometry

##### Apoptosis Assessment

Apoptosis was evaluated by flow cytometry using an eBioscience™ Annexin V (FITC/PI) apoptosis detection kit (Thermo Fisher Scientific, Waltham, MA, USA), in accordance with the manufacturer’s instructions. The seeding densities of both cell lines, H1299 and A549, along with the treatment protocol and analytical procedures, were conducted in accordance with the methodology previously described by Vlahova et al. [28]. The gating strategy was established on untreated control cells and applied consistently to all treatment conditions. The redistribution of treated cell populations toward the central region of the dot plots reflects the heterogeneous and progressive nature of the apoptotic response induced by **5d**.

##### Analysis of Cell Cycle Distribution

Cell cycle distribution was performed using a methodology consistent with that previously described in detail by Vlahova et al. [28], including cell seeding density, treatment conditions, sample preparation, and analytical procedures.

#### 2.2.5. Immunofluorescence Microscopy

Immunofluorescence staining was performed as previously described by Vlahova et al. [28], with minor modifications. Briefly, cells were seeded on glass coverslips (4 × 10^4^ cells per coverslip), allowed to adhere for 48 h, and treated as indicated. Following treatment, cells were fixed with 3.7% paraformaldehyde and methanol, permeabilized with 0.1% Triton X-100, and blocked in 3% BSA with 0.1% Tween 20 in PBS.

Primary antibodies against p53 (mouse monoclonal, 1:1000, Abcam, Cambridge, UK), phospho-histone H2AX (γH2AX, rabbit monoclonal, 1:200, Cell Signaling Technologies, Danvers MA, USA), and LC3B (rabbit polyclonal, 1:2000, Abcam, Cambridge, UK) were applied overnight at 4 °C. Secondary antibodies conjugated to Alexa Fluor dyes (Thermo Fisher Scientific, Waltham, MA, USA) were used at a dilution of 1:2000 for 30 min at room temperature.

EdU incorporation was assessed by incubation with 25 µM EdU for 30 min prior to fixation, followed by a click chemistry reaction using Alexa Fluor 488 azide (Thermo Fisher Scientific, Waltham, MA, USA). Caspase-3/7 (CellEvent Caspase-3/7 Green Detection Reagent (Thermo Fisher Scientific, Waltham, MA, USA)) activation was detected using a fluorescent probe according to the manufacturer’s instructions.

Coverslips were mounted with antifade medium containing DAPI (ProLong Diamond Antifade mounting media (Thermo Fisher Scientific, Waltham, MA, USA)), and fluorescence images were acquired using a Zeiss Axio Imager.A2 microscope (Carl Zeiss, Oberkochen, Germany) equipped with appropriate objectives (EC Plan-Neofluar 20×/0.5 M27 and Plan-Apochromat 63×/1.4 Oil DIC M27) and a CCD camera (Carl Zeiss, Oberkochen, Germany). All images were captured using identical exposure settings.

Image analysis was carried out using CellProfiler (version 4.2.6, Broad Institute’s Imaging Platform, Cambridge, MA, USA) and Fiji (ImageJ Software 2.15.1; https://hpc.nih.gov/apps/Fiji.html, accessed on 15 June 2026), and quantitative data were obtained from at least three independent experiments, with a minimum of 10 randomly selected fields analyzed per condition.

#### 2.2.6. Western Blot Analysis

Cell lysates were prepared and analyzed by Western blot as previously described [32], with minor modifications. Cells were lysed in ice-cold RIPA buffer (50 mM Tris-HCl, pH 7.5, 150 mM NaCl, 1% NP-40, 0.5% sodium deoxycholate, 0.1% SDS) supplemented with cOmplete™ EDTA-free protease inhibitor cocktail (Roche, Merck, Darmstadt, Germany). Lysates were sonicated briefly on ice and cleared by centrifugation at 7000× *g* for 15 min at 4 °C. Protein concentrations were determined using the Bradford assay.

Equal amounts of protein (20 µg per lane) were resolved on 12% SDS–PAGE gels and transferred to 0.45 µm nitrocellulose membranes (Bio-Rad, Hercules, CA, USA) using a wet transfer system (Bio-Rad, Hercules, CA, USA). Membranes were blocked for 1 h at room temperature in 5% BSA in TBST (Tris-buffered saline with 0.1% Tween-20). Primary antibodies against LC3 (rabbit polyclonal, 1:3000, Abcam, Cambridge, UK) and β-actin (mouse monoclonal, 1:2000, Sigma-Aldrich, Merck, Darmstadt, Germany) were diluted in 5% BSA in TBST and incubated overnight at 4 °C. After three washes with TBST (10 min each), membranes were incubated with HRP-conjugated goat anti-rabbit (Thermo Fisher Scientific, Waltham, MA, USA) or goat anti-mouse (Thermo Fisher Scientific, Waltham, MA, USA) secondary antibodies (1:10,000) for 1 h at room temperature. Following three additional TBST washes, bands were visualized using SuperSignal™ West Pico PLUS chemiluminescent substrate (Thermo Fisher Scientific, Waltham, MA, USA) and a C-DiGit^®^ Blot Scanner (LI-COR Biosciences, Lincoln, NE, USA). Band intensities were quantified by densitometry using Fiji (ImageJ Software 2.15.1; https://hpc.nih.gov/apps/Fiji.html, accessed on 15 June 2026) and normalized to β-actin. Membranes were either reprobed or run in parallel when required.

#### 2.2.7. Statistical Analysis

All experiments were conducted using a minimum of three independent biological replicates unless specified otherwise. Quantitative results are expressed as mean ± standard deviation (SD). Where appropriate, data were evaluated for normality using the Shapiro–Wilk test before statistical analysis, and parametric methods were applied accordingly.

For cytotoxicity studies (MTT assay), half-maximal inhibitory concentration (IC_50_) values were determined by nonlinear regression using a four-parameter logistic model. Curve fitting was performed using the “log (concentration) vs. normalized response (variable slope)” function in GraphPad Prism (version 8.0.1, GraphPad Software, Boston, MA, USA). The selectivity index (SI) was calculated as the ratio of IC_50_ values obtained in normal cells to those in cancer cells (SI = IC_50_ normal/IC_50_ cancer), with higher values indicating increased selectivity toward malignant cells.

For flow cytometric analyses, a minimum of 100,000 events per sample were recorded for cell cycle distribution, while apoptosis assessment using Annexin V/propidium iodide staining was based on at least 20,000 events per sample.

Quantitative immunofluorescence analyses (γH2AX, EdU, p53, LC3, and caspase-3/7) were performed in at least three independent experiments. For each condition, a minimum of 10 randomly selected microscopic fields per experiment were analyzed, ensuring the quantification of at least 100 cells per condition.

Statistical analyses were performed using one-way analysis of variance (ANOVA) followed by Tukey’s post hoc multiple comparisons test, implemented in R (version 4.3.2; R Foundation for Statistical Computing, Vienna, Austria), with RStudio (version 2026.04.0; Posit Software, Boston, MA, USA) used as the interface. A *p*-value below 0.05 was considered statistically significant. Group differences are indicated using lettering, where groups sharing at least one common letter are not significantly different (*p* > 0.05), whereas groups with distinct letters differ significantly (*p* < 0.05).

## 3. Results

### 3.1. Chemistry

**Scheme 1:**
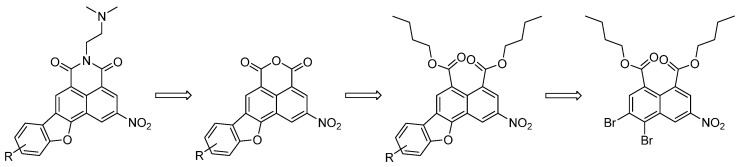
Retrosynthetic analysis of nitro-substituted benzofuran-fused naphthalimides.

Building on our previous studies on the synthesis of dioxin- and dibenzofuran-substituted naphthalimides [28,33], a synthetic strategy was developed to enable the introduction of nitro groups in both the naphthalimide and benzofuran moieties. The adopted approach utilized the previously reported by us 3,4-dibromo-6-nitro-NI [34] as starting material, and involved transformations via dibutyl esters, allowing the preparation of substituted anhydrides with high purity (Scheme 1).

**Scheme 2:**
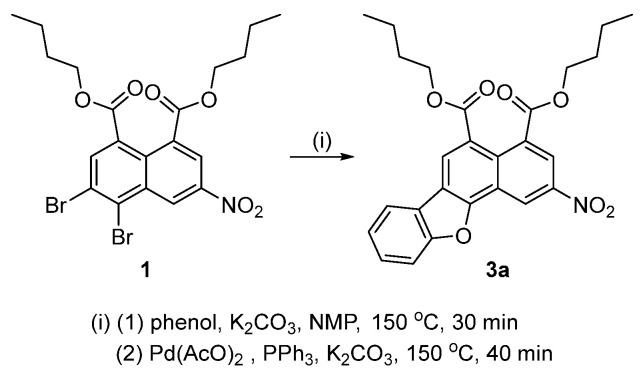
Direct one-pot synthesis of ester **3a** from dibutyl ester **1**.

For the synthesis of nitro-substituted benzofuran derivatives, previously optimized conditions for the preparation of dibenzofuran esters were applied [33]. The reaction of dibutyl ester **1** with phenol was carried out in NMP at 150 °C in the presence of potassium carbonate, leading to the complete conversion of the starting material to the corresponding 4-substituted intermediate within 2 h. Subsequently, palladium acetate and triphenylphosphine were added, and the reaction was conducted under an inert atmosphere. Complete consumption of the intermediate (monitored by TLC) was observed after approximately 2 h, affording the target ester (Scheme 2). After work-up and purification by column chromatography, ester **3a** was isolated in 43% yield. Its structure was confirmed by ^1^H and ^13^C NMR spectroscopy, and its purity was verified by elemental analysis.

Interestingly, the yield of this transformation was significantly lower compared to related systems, despite involving only a single C–H activation step [33]. This observation is likely related to the reduced stability of the nitro group on the naphthalimide core under strongly basic conditions. To improve the yield, a stepwise approach was investigated, involving the isolation of the intermediate followed by the optimization of the C–H activation step.

**Scheme 3:**
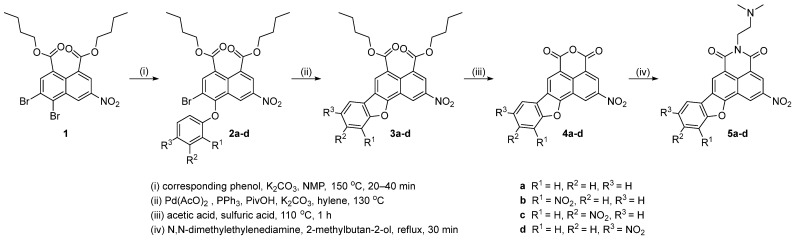
Synthesis of nitro-substituted benzofuran-fused naphthalimides.

Thus, the reaction of dibutyl ester **1** with phenol under the previously described conditions afforded the 4-phenoxy-substituted ester **2a** in 97% yield after purification (Scheme 3).

Ester **2a** was subsequently subjected to various C–H activation conditions to optimize the reaction outcome (Table 1). The results demonstrated that polar solvents such as NMP, DMA, and DMSO significantly decreased the reaction yield (Table 1, entries 1–8), whereas nonpolar solvents such as toluene and xylene provided substantially improved yields. The addition of 18-crown-6 enhanced the solubility of the base under these conditions. The highest yield (96%) was obtained in xylene, likely due to the higher reaction temperature achievable in this solvent.

To further investigate the effect of an additional nitro group in the benzofuran moiety, including its positional influence, the synthesis of dinitro-substituted esters was explored. Based on the findings described above, these transformations were also performed via a two-step protocol. In this manner, dinitro esters **3b**–**d** were obtained in overall yields of 90%, 89%, and 92%, respectively (Scheme 3). Notably, shorter reaction times were observed for both the nucleophilic substitution of nitrophenols and the subsequent C–H activation step compared to unsubstituted phenol. Esters **3b**–**d** were isolated as white solids, readily soluble in common organic solvents. The structures of dinitro esters **3b**–**d** were confirmed by ^1^H and ^13^C NMR spectroscopy, and their purity was verified by elemental analysis.

Conversion of esters **3a**–**d** to the corresponding anhydrides **4a**–**d** was achieved under identical conditions by heating in glacial acetic acid in the presence of concentrated sulfuric acid. In all cases, the anhydrides precipitated from the reaction mixture and were isolated by simple filtration followed by washing with methanol, affording the products in near-quantitative yields.

Subsequent imidization of anhydrides **4a**–**d** with *N*,*N*-dimethylethylenediamine was carried out in tert-amyl alcohol under reflux for 30 min, yielding the corresponding imides **5a**–**d** in high purity and near-quantitative yields.

### 3.2. Biology

#### 3.2.1. Cytotoxic Activity of Benzofuran-Containing Naphthalimide Derivatives

The cytotoxic activity of the synthesized benzofuran-containing naphthalimide derivatives was evaluated in human non-small-cell lung cancer cell lines A549 (p53 wild-type) and H1299 (p53-null), alongside non-malignant MRC-5 lung fibroblasts as a normal cell reference. The MTT assay was performed after 72 h of treatment. The obtained IC_50_ values are summarized in Table 2. All tested compounds exhibited potent antiproliferative activity in the nanomolar range, with a clear dependence on the benzofuran substitution pattern. Among the series, dinitro-substituted derivatives exhibited the highest potency. In particular, the 2,9-dinitro-substituted **5d** emerged as the most active compound, with IC_50_ values of 61 nM in A549 cells and 11 nM in H1299 cells. The mono-nitro derivative **5a** also showed strong cytotoxicity. The 2,11-dinitro analog **5b** was markedly less active, indicating a pronounced positional effect of nitro substituents on biological activity. The 2,10-dinitro-substituted analog **5c** exhibited high potency, but did not surpass that of **5d**. A comparison between the two cancer cell lines revealed increased sensitivity of the p53-deficient H1299 cells to the most potent compounds, especially **5d**.

The evaluated compounds exhibited favorable selectivity profiles toward malignant cells. The SI for **5d**, calculated from its IC_50_ of 352 nM in MRC-5 fibroblasts, was 5.8 in A549 cells and 32 in H1299 cells, indicating pronounced tumor selectivity, particularly in the p53-null context. Compound **5a** displayed a similarly favorable profile, with SI values of 7.7 for A549 and 11.5 for H1299. The 2,10-dinitro-substituted analog **5c** also exhibited good selectivity.

For comparison, the reference drugs doxorubicin and mitonafide had IC_50_ values of 242 nM and 644 nM, respectively, in A549 cells. In H1299 cells, their IC_50_ values were 120 nM and 490 nM [28]. Both drugs displayed substantially lower selectivity toward non-malignant cells. Notably, mitonafide showed an IC_50_ of 3064 nM in MRC-5 cells, reflecting moderate selectivity but comparatively lower absolute potency. The lead compound **5d** exhibited markedly higher cytotoxic potency than both reference drugs in both cell lines, with the difference being most pronounced in p53-null H1299 cells.

These findings demonstrate that incorporating a benzofuran moiety into the naphthalimide scaffold, particularly with dinitro substitution, results in markedly enhanced cytotoxic potency and tumor selectivity. The superior activity of compounds such as **5d** and **5c** highlights the critical role of substituent positioning in modulating biological activity.

#### 3.2.2. Doxorubicin- and Naphthalimide-Induced Inhibition of Clonogenic Survival

Clonogenic survival assays were performed on A549 and H1299 cells to assess long-term antiproliferative effects. The cells were treated with doxorubicin, mitonafide, and the lead compound **5d**.

**Figure 1:**
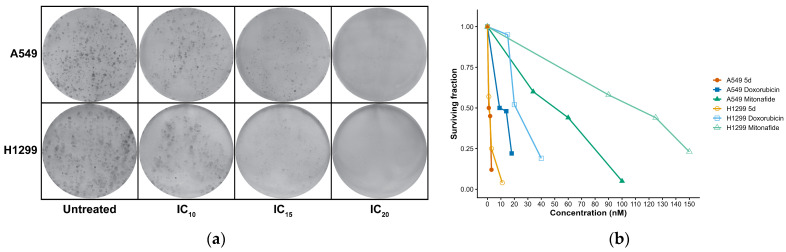
Inhibition of colony-forming ability by doxorubicin and naphthalimide derivatives. (**a**) Representative images of A549 and H1299 colonies obtained 10–14 days after exposure to **5d** at concentrations corresponding to IC_10_, IC_15_, and IC_20_; (**b**) quantitative assessment of clonogenic survival in A549 and H1299 cells, presented as surviving fractions relative to untreated controls.

Untreated control cells formed numerous dense, well-defined colonies, reflecting high proliferative capacity. Treatment with **5d** reduced both colony number and size in a dose-dependent manner (Figure 1a), with reductions evident even at the lowest concentrations tested (IC_10_ and IC_15_) and substantial suppression of clonogenic growth at IC_20_.

Quantitative analysis confirmed a significant decrease in the surviving fraction in both A549 and H1299 cells following treatment with **5d** (Figure 1b). This effect was more pronounced than that of the reference compounds, particularly in H1299 cells, where clonogenic survival was strongly impaired.

These results demonstrate that **5d** not only induces acute cytotoxicity but also durably suppresses the long-term proliferative potential of cancer cells, consistent with the induction of persistent replication stress and DNA damage.

#### 3.2.3. Effect of **5d** on Cell Cycle Progression

To characterize the mechanistic basis of **5d**-induced cytotoxicity, its effect on cell cycle progression was examined by propidium iodide staining and flow cytometry.

**Figure 2:**
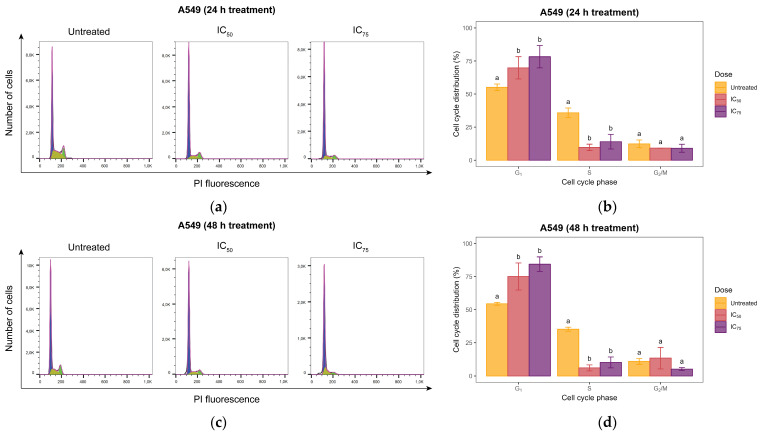
Effect of compound **5d** on cell cycle distribution in A549 cells, assessed by flow cytometry after 24 and 48 h of treatment at IC_50_ and IC_75_. (**a**,**c**) Representative PI fluorescence histograms. (**b**,**d**) Quantification of cell cycle distribution (G_1_, S, and G_2_/M phases), expressed as percentages of the total cell population. Data are presented as mean ± SD from three independent experiments. Comparisons were performed within each cell cycle phase across treatment conditions. Group differences are indicated using lettering, where groups sharing at least one common letter are not significantly different (*p* > 0.05), whereas different letters indicate significant differences (*p* < 0.05).

Treatment of A549 cells (p53 wild-type) with **5d** caused a marked accumulation of cells in the G_1_ phase and a corresponding decrease in S-phase cells at both 24 h and 48 h (Figure 2). This effect was more pronounced at higher concentrations (IC_75_) and after longer exposure, indicating a time- and dose-dependent G_1_ arrest. The increase in G_1_-phase cells is consistent with activation of a p53-dependent checkpoint following DNA damage.

**Figure 3:**
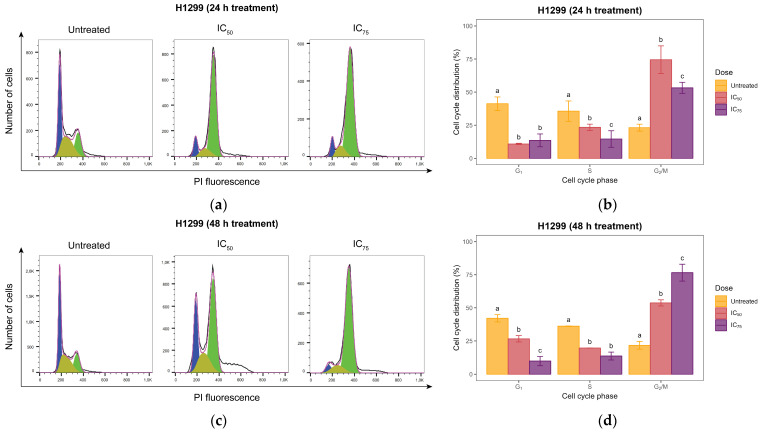
Effect of compound **5d** on cell cycle distribution in H1299 cells, assessed by flow cytometry after 24 and 48 h of treatment at IC_50_ and IC_75_. (**a**,**c**) Representative PI fluorescence histograms. (**b**,**d**) Quantification of cell cycle distribution (G_1_, S, and G_2_/M phases), expressed as percentages of the total cell population. Data are presented as mean ± SD from three independent experiments. Comparisons were performed within each cell cycle phase across treatment conditions. Group differences are indicated using lettering, where groups sharing at least one common letter are not significantly different (*p* > 0.05), whereas different letters indicate significant differences (*p* < 0.05).

In contrast, H1299 cells (p53-null) exhibited a distinct response, with progressive accumulation in the G_2_/M phase at both IC_50_ and IC_75_ and a concurrent reduction in the G_1_ population (Figure 3). This arrest became more pronounced after 48 h, consistent with the activation of the G_2_/M DNA damage checkpoint as a compensatory mechanism in the absence of functional p53, rendering these cells more dependent on G_2_/M checkpoint control to manage genotoxic stress.

In both cell lines, a reduction in the S-phase population was observed following treatment, indicating impaired DNA synthesis and providing initial evidence for replication stress induction.

Collectively, these results demonstrate differential cell cycle responses based on p53 status, with G_1_ arrest in A549 cells and G_2_/M arrest in H1299 cells. This divergence underscores the role of p53 in checkpoint activation and supports a mechanism of action involving DNA damage and replication stress, consistent with the EdU and γH2AX data presented below.

#### 3.2.4. Induction of Replication Stress and DNA Damage

To determine whether the antiproliferative activity of **5d** is associated with the disruption of DNA replication and induction of DNA damage, EdU incorporation and γH2AX immunofluorescence were assessed in A549 and H1299 cells treated at IC_50_ and IC_75_ concentrations for 24 and 48 h.

**Figure 4:**
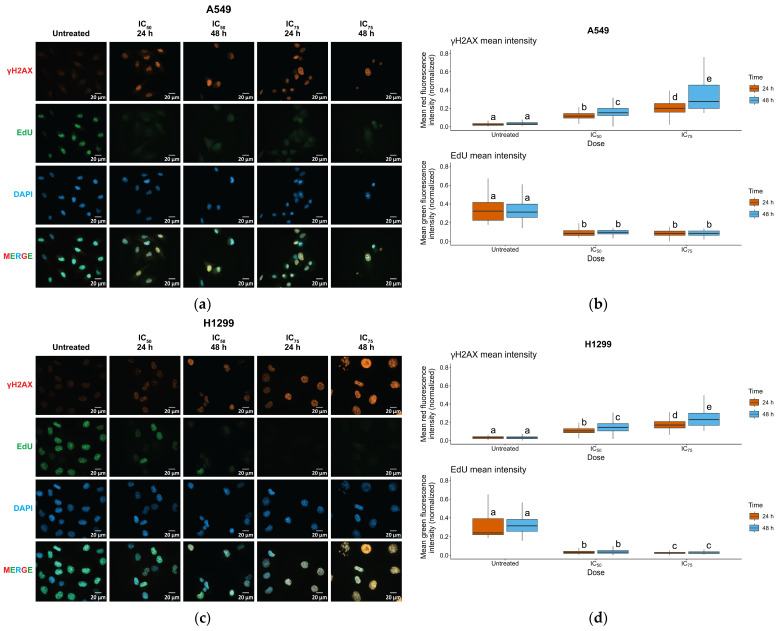
Replication stress and DNA damage induced by **5d** were evaluated in A549 and H1299 cells by immunofluorescence analysis of EdU incorporation and γH2AX foci formation after 24 and 48 h of treatment at IC_50_ and IC_75_ concentrations. (**a**,**c**) Representative immunofluorescence images showing γH2AX foci (red), EdU incorporation (green), and DAPI-stained nuclei (blue). Scale bar: 20 µm. (**b**,**d**) Quantification of γH2AX and EdU mean fluorescence intensities, expressed as the ratio of measured signal intensity to the corresponding nuclear area. Group differences are indicated using lettering, where groups sharing at least one common letter are not significantly different (*p* > 0.05), whereas different letters indicate significant differences (*p* < 0.05). *n* = 3 independent experiments.

Treatment with **5d** resulted in a significant concentration- and time-dependent increases in γH2AX signal intensity in both cell lines, with the most pronounced accumulation observed at IC_75_ after 48 h (Figure 4).

Concurrently, EdU incorporation was substantially reduced following **5d** treatment in both cell lines, with the greatest suppression observed at IC_75_ after 48 h, indicating a marked inhibition of active DNA synthesis.

The inverse relationship between EdU incorporation and γH2AX accumulation supports a replication stress-driven mechanism of DNA damage induction. Taken together, these results demonstrate that **5d** induces robust replication stress and DNA damage accumulation in both lung cancer cell lines, providing mechanistic support for its potent antiproliferative activity.

#### 3.2.5. Induction of Apoptosis by **5d**

**Figure 5:**
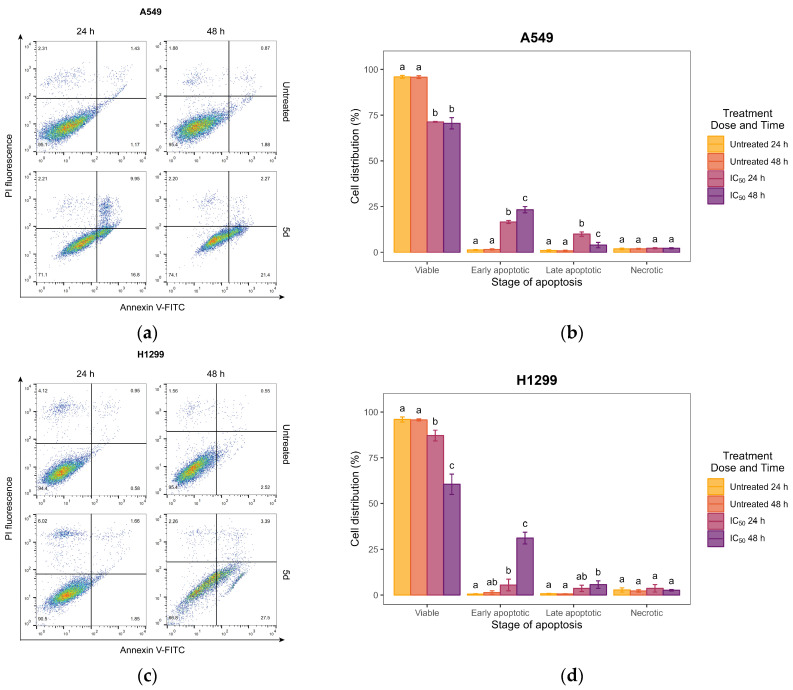
Induction of apoptosis by **5d** at IC_50_ concentrations in A549 and H1299 cells after 24 and 48 h of treatment. (**a**,**c**) Representative Annexin V–FITC/PI dot plots of cells following treatment with **5d.** (**b**,**d**) Quantitative distribution of cell populations expressed as percentages of viable (Annexin V^−^/PI^−^), early apoptotic (Annexin V^+^/PI^−^), late apoptotic (Annexin V^+^/PI^+^), and necrotic (Annexin V^−^/PI^+^) cells. Data represent mean ± SD from three independent experiments. Comparisons were performed within each cell population across treatment conditions. Group differences are indicated using lettering, where groups sharing at least one common letter are not significantly different (*p* > 0.05), whereas different letters indicate significant differences (*p* < 0.05).

To determine whether the cytotoxic effects of **5d** involve apoptotic cell death, Annexin V–FITC/PI staining and flow cytometry were performed in A549 and H1299 cells. In A549 cells, **5d** treatment resulted in a progressive reduction in the viable cell fraction and a corresponding increase in early and late apoptotic populations at both 24 h and 48 h, with the response becoming more pronounced over time (Figure 5). H1299 cells showed a comparable trend, with a marked decrease in viable cells and increased apoptosis following treatment. Early apoptosis increased in both cell lines at 24 and 48 h following treatment. However, the rise was modest in A549 cells, whereas H1299 cells showed a slight increase at 24 h followed by a pronounced elevation at 48 h, indicating a stronger time-dependent apoptotic response in the p53-null model.

These results confirm that apoptosis represents a major mode of cell death induced by **5d** in both cell lines, with the increase in Annexin V-positive populations consistent with caspase-3/7 activation, as further examined below.

#### 3.2.6. Activation of Executioner Caspases by **5d**

To confirm the engagement of the apoptotic execution machinery, caspase-3/7 activation was assessed by immunofluorescence in cells treated with **5d**.

**Figure 6:**
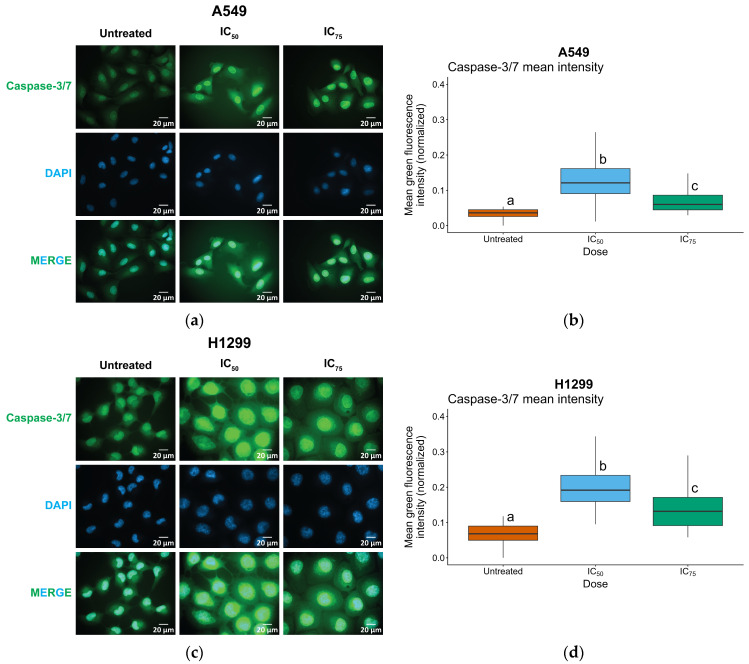
Caspase-3/7 activation in A549 and H1299 cells following 24 h treatment with **5d** at IC_50_ and IC_75_ concentrations. (**a**,**c**) Representative immunofluorescence images showing activated caspase-3/7 (green) and DAPI-stained nuclei (blue). Scale bar: 20 µm. (**b**,**d**) Quantitative analysis of caspase-3/7 mean fluorescence intensity, expressed as the ratio of measured signal intensity to the corresponding nuclear area. Group differences are indicated using lettering, where groups sharing at least one common letter are not significantly different (*p* > 0.05), whereas different letters indicate significant differences (*p* < 0.05). *n* = 3 independent experiments.

Treatment with **5d** resulted in a significant increase in caspase-3/7 activation in both A549 and H1299 cells compared with untreated controls (Figure 6). In both cell lines, caspase activation was more pronounced at IC_50_, while a reduction was observed at IC_75_, suggesting a potential shift toward late-stage apoptosis or secondary necrotic processes or altered kinetics of caspase activation at higher concentrations.

While an increase in caspase-3/7 activity was observed in both cell lines, the response was more pronounced in H1299 cells, whereas A549 cells exhibited a comparatively moderate activation, suggesting differences in apoptotic signaling that may be influenced by p53 status.

These results confirm the engagement of the caspase-dependent apoptotic program in response to **5d**, consistent with the DNA damage, replication stress, and cell cycle perturbations described above.

#### 3.2.7. Nuclear Accumulation of p53 in A549 Cells

**Figure 7:**
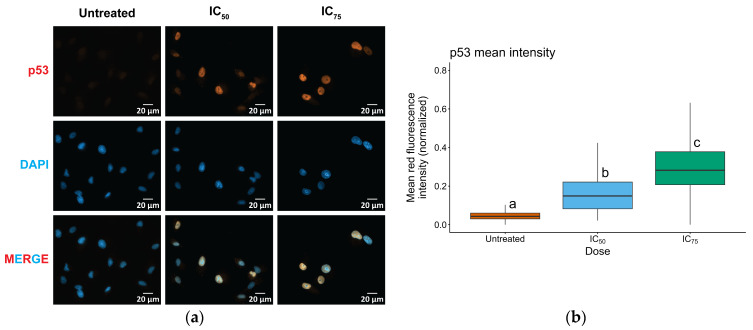
Nuclear accumulation of p53 in A549 cells following 24 h exposure to **5d** at IC_50_ and IC_75_ concentrations. (**a**) Representative immunofluorescence images showing p53 (red) with DAPI-stained nuclei (blue). Scale bar: 20 µm. (**b**) Quantitative analysis of nuclear p53 mean fluorescence intensity, expressed as the ratio of measured signal intensity to the corresponding nuclear area. Group differences are indicated using lettering, where groups sharing at least one common letter are not significantly different (*p* > 0.05), whereas different letters indicate significant differences (*p* < 0.05). *n* = 3 independent experiments.

To investigate p53 involvement in the cellular response to **5d**, immunofluorescence analysis of p53 localization was performed in A549 cells following 24 h treatment. A marked, dose-dependent increase in nuclear p53 signal intensity was observed in treated cells relative to untreated controls (Figure 7a), with greater accumulation at IC_75_ than at IC_50_. Quantitative analysis confirmed a significant increase in mean nuclear p53 fluorescence intensity (Figure 7b).

The nuclear accumulation of p53 is consistent with DNA damage induction and replication stress, and aligns with the G_1_ arrest observed in A549 cells, collectively supporting activation of a p53-mediated stress response. This effect is specific to A549 cells, which retain functional p53.

#### 3.2.8. Autophagy-Related LC3 Responses Following **5d** Treatment

To further investigate the cellular response to **5d**, the induction of activation of autophagy-related processes was assessed by immunofluorescence analysis of LC3-positive puncta formation.

**Figure 8:**
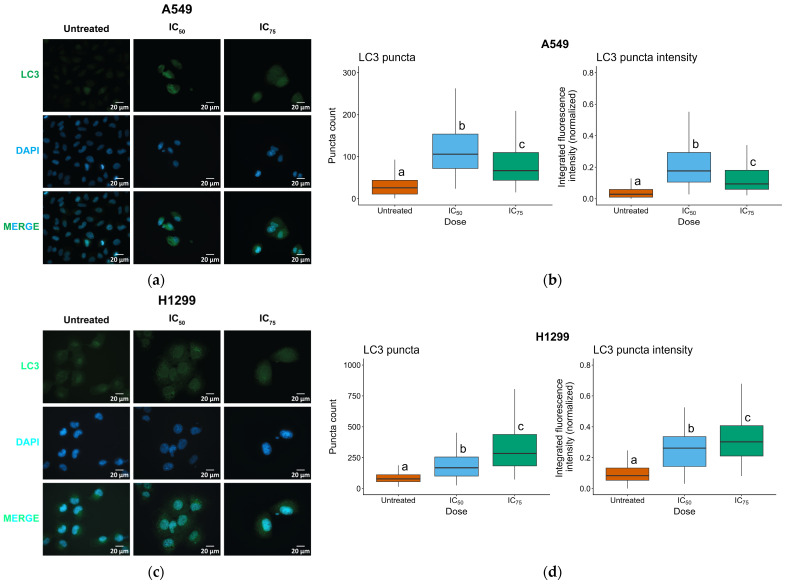
Autophagy-related LC3 responses following **5d** treatment in A549 and H1299 cells following 24 h treatment with **5d** at IC_50_ and IC_75_ concentrations. (**a**,**c**) Representative immunofluorescence images of LC3 puncta formation (green), with DAPI-stained nuclei (blue). Scale bar: 20 µm. (**b**,**d**) Quantification of the average number of LC3-positive puncta per cell and LC3 puncta intensity. Group differences are indicated using lettering, where groups sharing at least one common letter are not significantly different (*p* > 0.05), whereas different letters indicate significant differences (*p* < 0.05). *n* = 3 independent experiments.

Treatment with **5d** led to a clear increase in LC3-positive structures in both A549 and H1299 cells compared with untreated controls (Figure 8a,c). These punctate LC3 signals, consistent with autophagosome accumulation, were more prominent at higher concentrations (IC_75_), suggesting a dose-dependent enhancement of autophagy-related LC3 redistribution in H1299 cells.

Quantitative analysis confirmed a significant increase in both the number and average intensity of LC3-positive granules per cell in both cell lines (Figure 8b,d), further supporting LC3 redistribution.

The autophagy-related LC3 response was more pronounced in H1299 cells, consistent with their greater sensitivity to **5d** and the stronger replication stress phenotype observed in this cell line.

**Figure 9:**
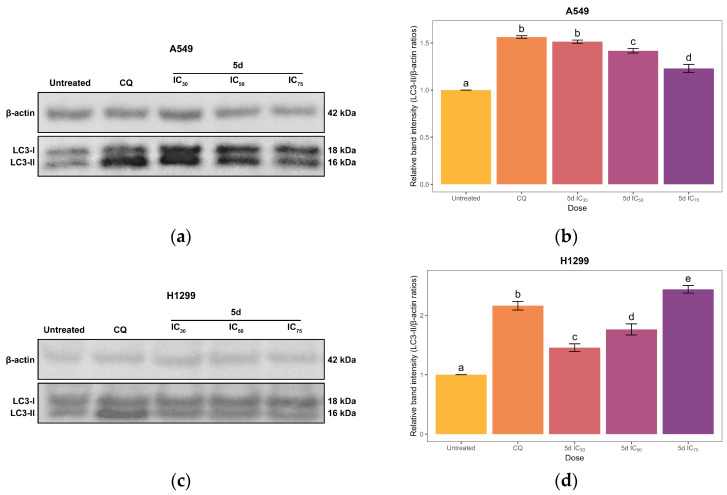
LC3 expression in A549 and H1299 cells after 24 h treatment with IC_30_, IC_50_, and IC_75_ of **5d**. (**a**,**c**) Western blots for LC3 with β-actin used as a loading control and chloroquine (CQ, 50 µM) as a positive control. (**b**,**d**) Quantification of LC3-II expression levels. Data represent mean ± SD from three independent experiments. Group differences are indicated using lettering, where groups sharing at least one common letter are not significantly different (*p* > 0.05), whereas different letters indicate significant differences (*p* < 0.05).

To further support the autophagy-related LC3 responses following **5d** treatment and evaluate LC3 processing, LC3-I to LC3-II conversion was examined by Western blot analysis in both cell lines following 24 h treatment with **5d** at IC_30_, IC_50_, and IC_75_ concentrations, using chloroquine (CQ, 50 µM) as a positive control (Figure 9). Treatment with **5d** resulted in a concentration-dependent increase in LC3-II levels in H1299 cells, with the highest accumulation observed at IC_75_, consistent with the immunofluorescence data. In A549 cells, LC3-II levels were most pronounced at IC_30_ and showed an inverse relationship with increasing concentration, suggesting that at higher doses, apoptotic cell death becomes the predominant cellular response, potentially limiting sustained autophagic activity.

The observed accumulation of LC3-II, together with the increased formation of LC3-positive puncta, supports the activation of autophagy-related processes in response to **5d** treatment. However, because dedicated flux experiments were not performed, the present findings should be interpreted as evidence of enhanced LC3 processing and autophagosome accumulation rather than definitive proof of increased autophagic flux. These observations suggest cell context-dependent differences in autophagy-related responses.

## 4. Discussion

Among the synthesized benzofuran-containing naphthalimide derivatives, the 2,9-dinitro-substituted **5d** emerged as the most potent and selective compound. Its antitumor activity is mediated through a coordinated cellular response encompassing the inhibition of DNA synthesis, accumulation of DNA damage, checkpoint activation, and induction of apoptotic cell death. This mechanistic profile is consistent with the established behavior of DNA-targeting naphthalimides and with the concept that cancer cells, which operate under elevated endogenous replication stress, are particularly vulnerable to agents that further impair replication fork progression [28,35].

The biological activity of **5d** is supported by a convergent set of experimental evidence. In addition to demonstrating nanomolar cytotoxicity, **5d** significantly reduced clonogenic survival, indicating that its effects extend beyond short-term metabolic inhibition to a durable loss of proliferative capacity. This distinction is notable, as clonogenic assays provide a more rigorous assessment of long-term antitumor efficacy compared to acute viability assays [36,37]. This sustained antiproliferative effect is consistent with persistent replication-associated DNA lesions and impaired recovery of proliferative potential following treatment.

Cell cycle data further support this interpretation and reveal a clear divergence between the two lung cancer models. In A549 cells, which retain functional p53, **5d** induced G_1_ phase accumulation, whereas p53-deficient H1299 cells predominantly exhibited G_2_/M arrest. This pattern is consistent with canonical checkpoint biology: cells with intact p53 can engage a G_1_ checkpoint following genotoxic stress, while p53-deficient cells are more dependent on S/G_2_ and G_2_/M checkpoint mechanisms for damage control. The dose-dependent nuclear accumulation of p53 in A549 cells further confirms that **5d** engages the p53 stress-response pathway in cells where it is functionally intact. The heightened sensitivity of p53-deficient H1299 cells to **5d** is consistent with the notion that impaired G_1_ checkpoint control renders these cells more susceptible to replication stress-inducing agents, as they are unable to engage a timely G_1_ arrest and must instead rely on S/G_2_ and G_2_/M checkpoint mechanisms to manage genotoxic insult.

The inverse relationship between EdU incorporation and γH2AX accumulation provides a key mechanistic insight: reduced EdU signal reflects impaired DNA synthesis, while elevated γH2AX levels mark the accumulation of DNA double-strand breaks arising from stalled or collapsed replication forks. Together, these findings support a replication stress-centered mechanism of action, clearly distinguishing **5d** from nonspecific cytotoxic agents. This interpretation aligns with current models of oncogene- and therapy-induced replication stress, in which the disruption of fork progression culminates in DNA break accumulation, checkpoint activation, and cell death [27].

These findings are consistent with the established pharmacology of naphthalimide derivatives, whose anticancer effects are closely linked to DNA intercalation, alteration in DNA topology, and topoisomerase-associated damage responses [16,17]. Although the direct molecular target of **5d** has not been identified, the observed cellular phenotype—characterized by replication stress, DNA damage accumulation, and checkpoint activation—is consistent with DNA-targeting, topoisomerase-perturbing agents.

The present benzofuran–naphthalimide series differs from classical antireplicative agents such as hydroxyurea and gemcitabine, which exert their effects primarily through the direct inhibition of ribonucleotide reductase or nucleotide incorporation, respectively, without a significant DNA intercalation component [4,38]. In contrast, the naphthalimide core of **5d** is expected to engage DNA through intercalation and topoisomerase perturbation, while the extended benzofuran system and nitro substituents modulate electronic properties and reactive oxygen species generation, together producing a multi-modal mechanism of replication stress induction. This mechanistic distinction may be relevant for the activity profile observed in p53-null cells, where dependence on S/G_2_ checkpoint pathways renders them particularly vulnerable to agents that combine intercalation with replication fork stalling.

Comparison with the recently reported benzodioxin-annulated naphthalimide series is particularly instructive [28]. The lead benzodioxin analog similarly demonstrated potent nanomolar cytotoxicity, inhibition of DNA synthesis, γH2AX induction, and a p53-dependent divergence in cell cycle response, with G_1_ arrest in A549 cells and accumulation of H1299 cells in G_2_/M. The close similarity between these profiles supports the notion that both annulated scaffolds share a replication stress-driven mechanism of action. The current benzofuran series further demonstrates that structural variation in the annulated heterocycle and the positioning of nitro substituents can substantially influence potency and selectivity, underscoring the sensitivity of biological activity to structural modification within this compound class.

Benzofuran is regarded as a privileged scaffold in medicinal chemistry, and its rigid, conjugated character is known to promote interactions with DNA and enhance target engagement [21,22]. Within the present series, the superior activity of **5d** relative to both the mono-nitro analog and the positional dinitro isomers demonstrates that not only the presence but also the spatial arrangement of nitro substituents within the annulated system is critical for biological activity. These findings reveal a clear dependence of replication stress-inducing capacity on the electronic properties of the scaffold, providing a rational basis for further structural optimization.

The apoptosis data place the replication stress phenotype within a coherent mechanistic framework [39]. Annexin V/PI staining revealed a progressive increase in apoptotic populations, while caspase-3/7 activation confirmed the engagement of the execution phase of apoptosis. The pronounced apoptotic response in H1299 cells is consistent with their increased sensitivity to **5d** and suggests that p53-deficient cells, which cannot initiate G_1_ arrest, may be more vulnerable to the consequences of unresolved replication-associated DNA lesions. These results collectively identify apoptosis as the predominant cell death mechanism triggered by **5d**.

The detection of LC3-positive puncta together with concentration-dependent LC3-II accumulation supports the activation of autophagy-associated processes in response to **5d** in both cell lines. The differential concentration–response pattern observed between A549 and H1299 cells is particularly noteworthy: whereas H1299 cells exhibited a dose-dependent increase in LC3-II levels peaking at IC_75_, A549 cells showed the most pronounced LC3-II accumulation at IC_30_, with an inverse relationship at higher concentrations. This divergence likely reflects a shift toward apoptosis as the dominant cellular outcome at higher doses in A549 cells, where the intact p53 pathway may accelerate commitment to apoptosis and limit sustained autophagy-related LC3 responses. In p53-deficient H1299 cells, the sustained autophagy-related response across a broader concentration range may reflect altered stress adaptation in the absence of p53-mediated apoptotic signaling. This interpretation is consistent with evidence that p53 status influences the balance between autophagy and apoptosis under genotoxic stress, with p53-deficient cells often exhibiting prolonged autophagy-related responses prior to cell death [40]. Given the pronounced replication stress, DNA damage, and apoptosis observed across all experimental conditions, autophagy most likely represents an adaptive stress response rather than a primary mechanism of cell death. Because dedicated autophagic flux experiments were not performed, the present findings should be interpreted as evidence of enhanced LC3 processing and autophagosome accumulation rather than definitive proof of increased flux. Future studies employing tandem fluorescent LC3 reporters or lysosomal inhibitors will be required to fully characterize the autophagy-related LC3 response to **5d**.

Notably, benzofuran-containing compounds have previously been reported to modulate autophagic pathways in cancer cells—Moracin N, a natural benzofuran derivative, was shown to induce autophagy and apoptosis in A549 lung cancer cells through ROS generation and mTOR inhibition, with autophagy serving as a pro-death mechanism [41]. In the context of the present study, the autophagy-related LC3 response observed following **5d** treatment may therefore reflect both a consequence of replication stress-induced DNA damage and pharmacological effects of the benzofuran moiety on autophagic regulatory pathways. Future mechanistic studies will be required to delineate these contributions.

The heightened activity of **5d** in p53-null H1299 cells has potential therapeutic implications. Tumors with defective p53 signaling frequently exhibit altered checkpoint architecture and greater dependence on non-G_1_ checkpoint pathways for survival under replication stress [42]. Agents that exploit this vulnerability represent an important therapeutic opportunity, as they may offer enhanced efficacy in tumors that are refractory to therapies relying on intact p53-mediated responses. The present findings therefore suggest that benzofuran-annulated naphthalimides may be of particular value in the treatment of checkpoint-defective, p53-null malignancies.

**Figure 10:**
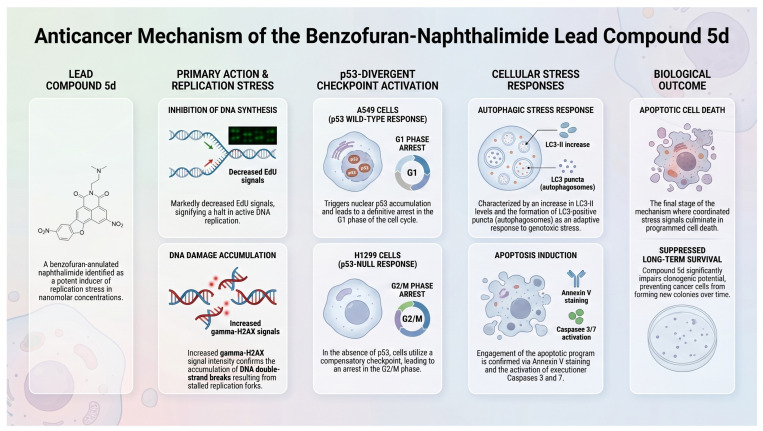
Graphical abstract. The benzofuran-containing naphthalimide **5d** induces replication stress, leading to the inhibition of DNA synthesis, DNA damage accumulation, checkpoint activation, and p53-dependent or independent cell cycle arrest. These events culminated in apoptosis and reduced long-term survival, accompanied by an autophagy-related stress response.

In summary, **5d** acts as a potent inducer of replication stress, suppressing DNA synthesis and promoting DNA damage accumulation in a manner that differentially engages checkpoint pathways depending on p53 status, ultimately culminating in apoptotic cell death with an associated autophagic stress response (Figure 10). Taken together with previous findings on benzodioxin-annulated naphthalimides [28], these results reinforce the concept that annulated naphthalimide scaffolds represent a versatile platform for the development of replication stress-oriented anticancer agents. Future studies should address the molecular mechanisms linking structural features to the observed cellular phenotypes, including the direct assessment of DNA binding affinity, topoisomerase inhibitory activity, and effects on replication fork dynamics.

## 5. Conclusions

In conclusion, **5d** emerges as a potent benzofuran-annulated naphthalimide that induces replication stress and triggers a coordinated cellular response leading to apoptotic cell death. Its nanomolar potency, favorable tumor selectivity, and p53-dependent mechanistic profile underscore the therapeutic relevance of this scaffold. These findings establish benzofuran-annulated naphthalimides as a promising platform for the development of next-generation replication stress-targeting anticancer agents.

## Figures and Tables

**Table 1:** Optimization of the C–H activation conditions for the synthesis of ester **3a**.

Entry	Solvent	Catalytic System *	Temperature (°C)	Base	Additives	Yield (%) **
1	NMP	Pd(OAc)_2_/PPh_3_	150	K_2_CO_3_	-	43
2	NMP	Pd(OAc)_2_/PPh_3_	120	K_2_CO_3_	-	50
3	NMP	Pd(OAc)_2_/PPh_3_	120	Cs_2_CO_3_	-	traces
4	NMP	Pd(OAc)_2_/PPh_3_	120	K_2_CO_3_	pivalic acid	51
5	NMP	Pd(OAc)_2_/PCy_3_	120	K_2_CO_3_	-	42
6	DMA	Pd(OAc)_2_/PCy_3_	120	K_2_CO_3_	-	48
7	DMA	Pd(OAc)_2_/PPh_3_	120	K_2_CO_3_	-	45
8	DMSO	Pd(OAc)_2_/PPh_3_	120	K_2_CO_3_	-	36
**9**	**xylene**	**Pd(OAc)_2_/PPh_3_**	**130**	**K_2_CO_3_**	**pivalic acid,** **18-crown-6**	**96**
10	toluene	Pd(OAc)_2_/PPh_3_	110	K_2_CO_3_	pivalic acid, 18-crown-6	87

* In all cases, a palladium source-to-ligand ratio of 1:2.5 was employed. ** All reactions were carried out at a 2 mmol scale, in 15 mL of solvent and 3 eq (6 mmol) of base.

**Table 2:** IC_50_ values (nM) for doxorubicin, mitonafide, and benzofuran–naphthalimides were determined, with results presented as mean ± SD from a minimum of three independent experiments. (see Figure S29 for detailed dose–response curves of benzofuran–naphthalimides).

Compound	A549		H1299		MRC-5
	IC_50_ ± SD	SI	IC_50_ ± SD	SI	IC_50_ ± SD
doxorubicin	242 ± 3.8	1.8	120 ± 9.5	3.6	441 ± 3.4
mitonafide	644 ± 4.1	4.8	490 ± 2.3	6.2	3064 ± 4.8
**5a**	82 ± 2.5	7.7	55 ± 4.9	11.5	630 ± 9.4
**5b**	213 ± 3.5	3.9	321 ± 1.9	2.6	832 ± 2.2
**5c**	93 ± 1.7	2.3	28 ± 8.1	7.6	213 ± 4.9
**5d**	61 ± 5.9	5.8	11 ± 1.7	32	352 ± 4.6

## Data Availability

The original contributions presented in this study are included in the article/Supplementary Materials. Further inquiries can be directed to the corresponding authors.

## Supplementary Materials

The following supporting information can be downloaded at: https://www.mdpi.com/article/10.3390/pharmaceutics18060754/s1, Figure S1: ^1^H NMR spectrum of the compound **2a** in chloroform-*d*; Figure S2: ^13^C NMR spectrum of the compound **2a** in chloroform-*d*; Figure S3: ^1^H NMR spectrum of the compound **2b** in chloroform-*d*; Figure S4: ^13^C NMR spectrum of the compound **2b** in chloroform-*d*; Figure S5: ^1^H NMR spectrum of the compound **2c** in chloroform-*d*; Figure S6: ^13^C NMR spectrum of the compound **2c** in chloroform-*d*; Figure S7: ^1^H NMR spectrum of the compound **2d** in chloroform-*d*; Figure S8: ^13^C NMR spectrum of the compound **2d** in chloroform-*d*; Figure S9: ^1^H NMR spectrum of the compound **3a** in chloroform-*d*; Figure S10: ^13^C NMR spectrum of the compound **3a** in chloroform-*d*; Figure S11: ^1^H NMR spectrum of the compound **3b** in chloroform-*d*; Figure S12: ^13^C NMR spectrum of the compound **3b** in chloroform-*d*; Figure S13: ^1^H NMR spectrum of the compound **3c** in chloroform-*d*; Figure S14: ^13^C NMR spectrum of the compound **3c** in chloroform-*d*; Figure S15: ^1^H NMR spectrum of the compound **3d** in chloroform-*d*; Figure S16: ^13^C NMR spectrum of the compound **3d** in chloroform-*d*; Figure S17: ^1^H NMR spectrum of the compound **5a** in trifluoroacetic acid-*d*; Figure S18: ^13^C NMR spectrum of the compound **5a** in trifluoroacetic acid-*d*; Figure S19: ^1^H NMR spectrum of the compound **5b** in trifluoroacetic acid-*d*; Figure S20: ^13^C NMR spectrum of the compound **5b** in trifluoroacetic acid-*d*; Figure S21: ^1^H NMR spectrum of the compound **5c** in trifluoroacetic acid-*d*; Figure S22: ^13^C NMR spectrum of the compound **5c** in trifluoroacetic acid-*d*; Figure S23: ^1^H NMR spectrum of the compound **5d** in trifluoroacetic acid-*d*; Figure S24: ^13^C NMR spectrum of the compound **5d** in trifluoroacetic acid-*d*; Figure S25: HRMS spectrum of compound **5a**; Figure S26: HRMS spectrum of compound **5b**; Figure S27: HRMS spectrum of compound **5c**; Figure S28: HRMS spectrum of compound **5d**; Figure S29: Dose–response curves of A549, H1299, and MRC-5 cells following 72 h exposure to different concentrations of compounds **5a** (a), **5b** (b), **5c** (c), and **5d** (d); Figure S30: Magnified views of representative cells from immunofluorescence analysis showing γH2AX foci (red), EdU incorporation (green), and DAPI-stained nuclei (blue) after 24 and 48 h treatment with **5d** at IC_50_ and IC_75_ concentrations in A549 (a) and H1299 (b) cells; Figure S31: Magnified views of representative cells showing LC3 puncta formation (green) and DAPI-stained nuclei (blue) after 24 h treatment with **5d** at IC_50_ and IC_75_ concentrations in A549 (a) and H1299 (b) cells.

## Abbreviations

The following abbreviations are used in this manuscript:

ATR	Ataxia Telangiectasia and Rad3-related protein
ANOVA	Analysis of variance
ATCC	American Type Culture Collection
BSA	Bovine serum albumin
CHK1	Checkpoint kinase 1
DAPI	4′,6-diamidino-2-phenylindole
DDR	DNA damage response
DMSO	Dimethyl sulfoxide
DSB	DNA double-strand break
EdU	5-ethynyl-2′-deoxyuridine
FITC	Fluorescein isothiocyanate
IC_50_	Half-maximal inhibitory concentration
LC3	Microtubule-associated protein 1A/1B-light chain 3
MTT	3-(4,5-dimethylthiazol-2-yl)-2,5-diphenyltetrazolium bromide
NI	Naphthalimide
PE	Plating efficiency
SF	Surviving fraction
SI	Selectivity index
γH2AX	Phosphorylated histone H2AX (Ser139)
