# Supplementary material for: Benzofuran-Annulated Naphthalimides Trigger Replication Stress, DNA Damage, and p53-Dependent Cell Cycle Arrest
Source: Pharmaceutics. 2026 Jun 20;18(6):754. doi: 10.3390/pharmaceutics18060754 (PMC13306732; doi:10.3390/pharmaceutics18060754)
Supplement: Supplementary file 1 [file pharmaceutics-18-00754-s001.zip › pharmaceutics-4348453-supplementary.pdf]

Article

Benzofuran-Annulated Naphthalimides Trigger Replication Stress, DNA Damage, and p53-Dependent Cell Cycle Arrest

Zlatina Vlahova [1], Lazar Lazarov [1], Maria Petrova [1], Shazie Yusein-Myashkova [1], Jordana Todorova [1], Maria Schröder [1], Monika Mutovska [2], Stanimir Stoyanov [2], Yulian Zagranyarski [2*], and Iva Ugrinova [1*]

[1] Institute of Molecular Biology "Akad. Roumen Tsanev", Bulgarian Academy of Sciences, Acad. G. Bonchev Str., bl. 21, 1113 Sofia, Bulgaria; vlahova94@gmail.com (Z.V.); lazarutko@gmail.com (L.L.); mhristova84@abv.bg (M.P.); shazi@abv.bg (S.Y.-M.); jordanabg@yahoo.com (J.T.); marias82@abv.bg (M.S.).

[2] Faculty of chemistry and pharmacy, Sofia University "St. Kliment Ohridski", 1 J. Baurchier blvd, 1164 Sofia, Bulgaria; ohmgm@chem.uni-sofia.bg (M.M), ohss@chem.uni-sofia.bg (S.S.).

* Correspondence: ohjz@chem.uni-sofia.bg (Y.Z.), ugryiva@gmail.com (I.U.);

Supplementary Information

Table of Contents

Figure S1: ^{1}H NMR spectrum of the compound **2a** in chloroform-*d* 3

Figure S2: ^{13}C NMR spectrum of the compound **2a** in chloroform-*d* 3

Figure S3: ^{1}H NMR spectrum of the compound **2b** in chloroform-*d* 4

Figure S4: ^{13}C NMR spectrum of the compound **2b** in chloroform-*d* 4

Figure S5: ^{1}H NMR spectrum of the compound **2c** in chloroform-*d* 5

Figure S6: ^{13}C NMR spectrum of the compound **2c** in chloroform-*d* 5

Figure S7: ^{1}H NMR spectrum of the compound **2d** in chloroform-*d* 6

Figure S8: ^{13}C NMR spectrum of the compound **2d** in chloroform-*d* 6

Figure S9: ^{1}H NMR spectrum of the compound **3a** in chloroform-*d* 7

Figure S10: ^{13}C NMR spectrum of the compound **3a** in chloroform-*d* 7

Figure S11: ^{1}H NMR spectrum of the compound **3b** in chloroform-*d* 8

Figure S12: ^{13}C NMR spectrum of the compound **3b** in chloroform-*d* 8

Figure S13: ^{1}H NMR spectrum of the compound **3c** in chloroform-*d* 9

Figure S14: ^{13}C NMR spectrum of the compound **3c** in chloroform-*d* 9

Figure S15: ^{1}H NMR spectrum of the compound **3d** in chloroform-*d* 10

Figure S16: ^{13}C NMR spectrum of the compound **3d** in chloroform-*d* 10

Figure S17: ^{1}H NMR spectrum of the compound **5a** in trifluoroacetic acid-*d* 11

Figure S18: ^{13}C NMR spectrum of the compound **5a** in trifluoroacetic acid-*d* 11

Figure S19: ^{1}H NMR spectrum of the compound **5b** in trifluoroacetic acid-*d* 12

Figure S20: ^{13}C NMR spectrum of the compound **5b** in trifluoroacetic acid-*d* 12

Figure S21: ^{1}H NMR spectrum of the compound **5c** in trifluoroacetic acid-*d* 13

Figure S22: ^{13}C NMR spectrum of the compound **5c** in trifluoroacetic acid-*d* 13

Figure S23: ^{1}H NMR spectrum of the compound **5d** in trifluoroacetic acid-*d* 14

Figure S24: ^{13}C NMR spectrum of the compound **5d** in trifluoroacetic acid-*d* 14

Figure S25: HRMS spectrum of compound **5a** 15

Figure S26: HRMS spectrum of compound **5b** 15

Figure S27: HRMS spectrum of compound **5c** 16

Figure S28: HRMS spectrum of compound **5d** 16

Figure S29: Dose–response curves of A549, H1299, and MRC-5 cells following 72 h exposure to different concentrations of compounds **5a** (a), **5b** (b), **5c** (c), and **5d** (d). 17

Figure S30: Magnified views of representative cells from immunofluorescence analysis showing γH2AX foci (red), EdU incorporation (green), and DAPI-stained nuclei (blue) after 24 and 48 h treatment with **5d** at IC_{50} and IC_{75} concentrations in A549 (a) and H1299 (b) cells. 17

Figure S31: Magnified views of representative cells showing LC3 puncta formation (green) and DAPI-stained nuclei (blue) after 24 h treatment with **5d** at IC_{50} and IC_{75} concentrations in A549 (a) and H1299 (b) cells 18

Figure S1: ^{1}H NMR spectrum of the compound **2a** in chloroform-*d*

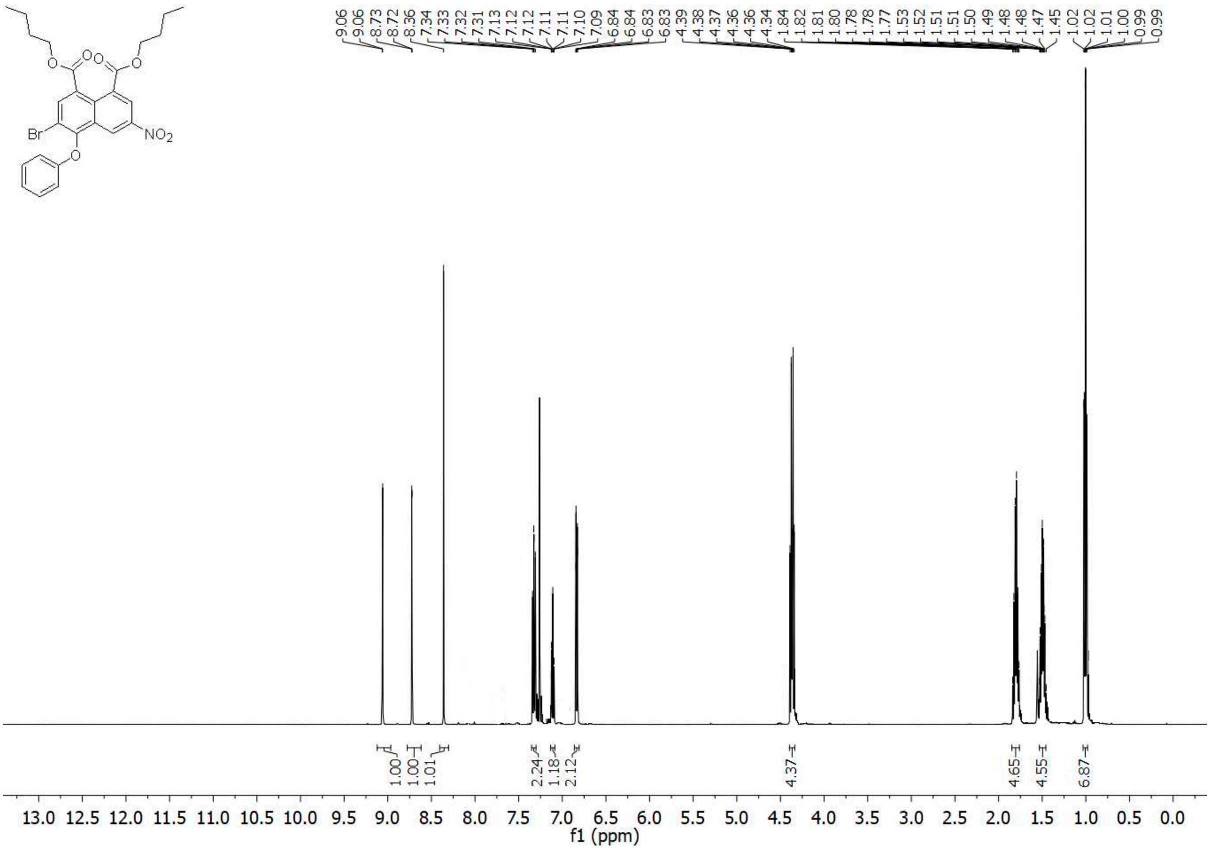

Figure S2: ^{13}C NMR spectrum of the compound **2a** in chloroform-*d*

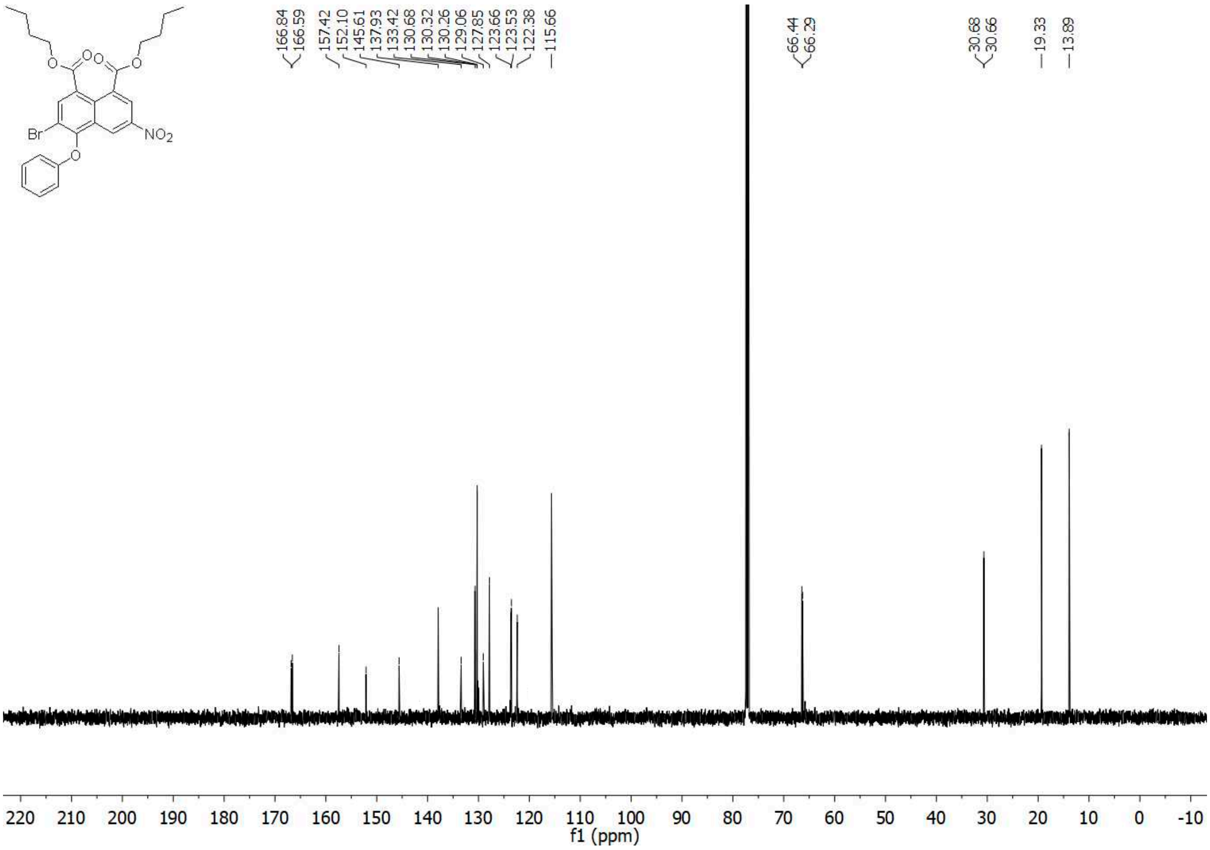

Figure S3: ^{1}H NMR spectrum of the compound **2b** in chloroform-*d*

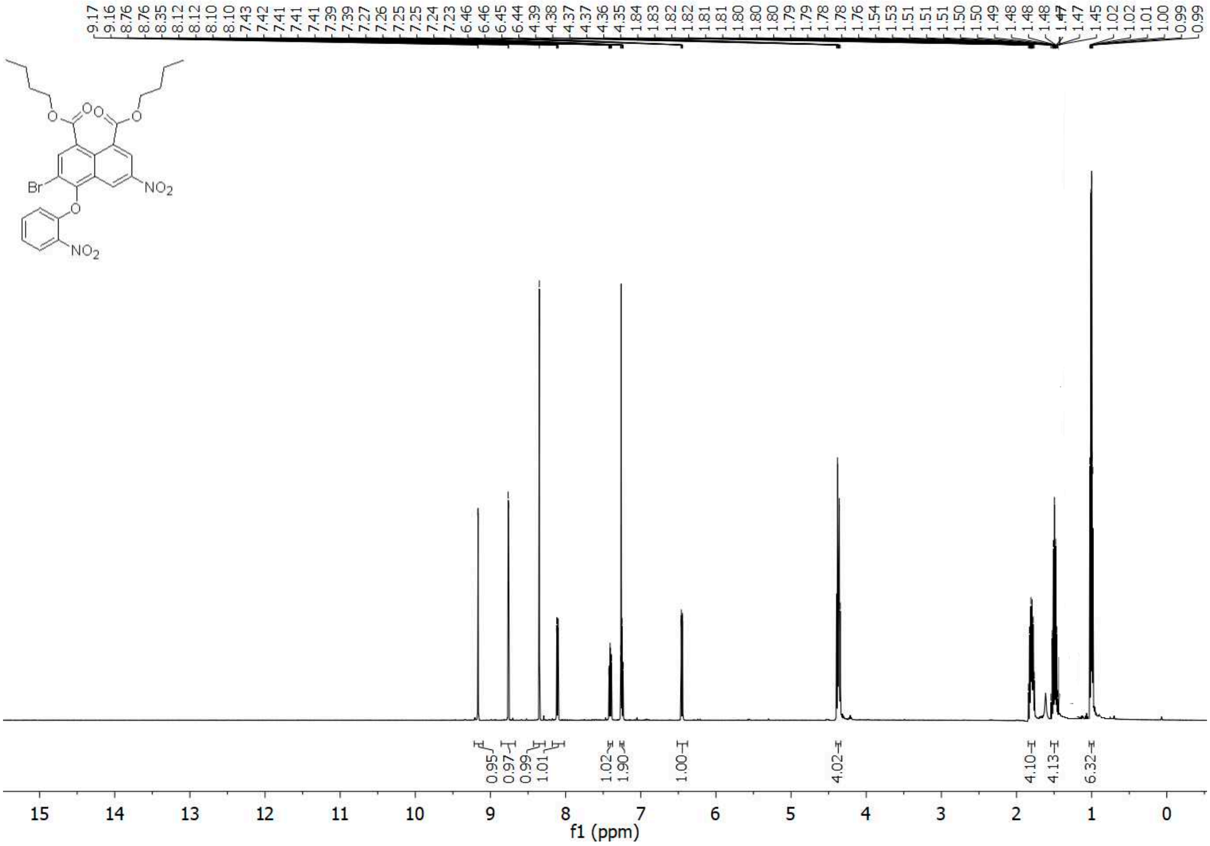

Figure S4: ^{13}C NMR spectrum of the compound **2b** in chloroform-*d*

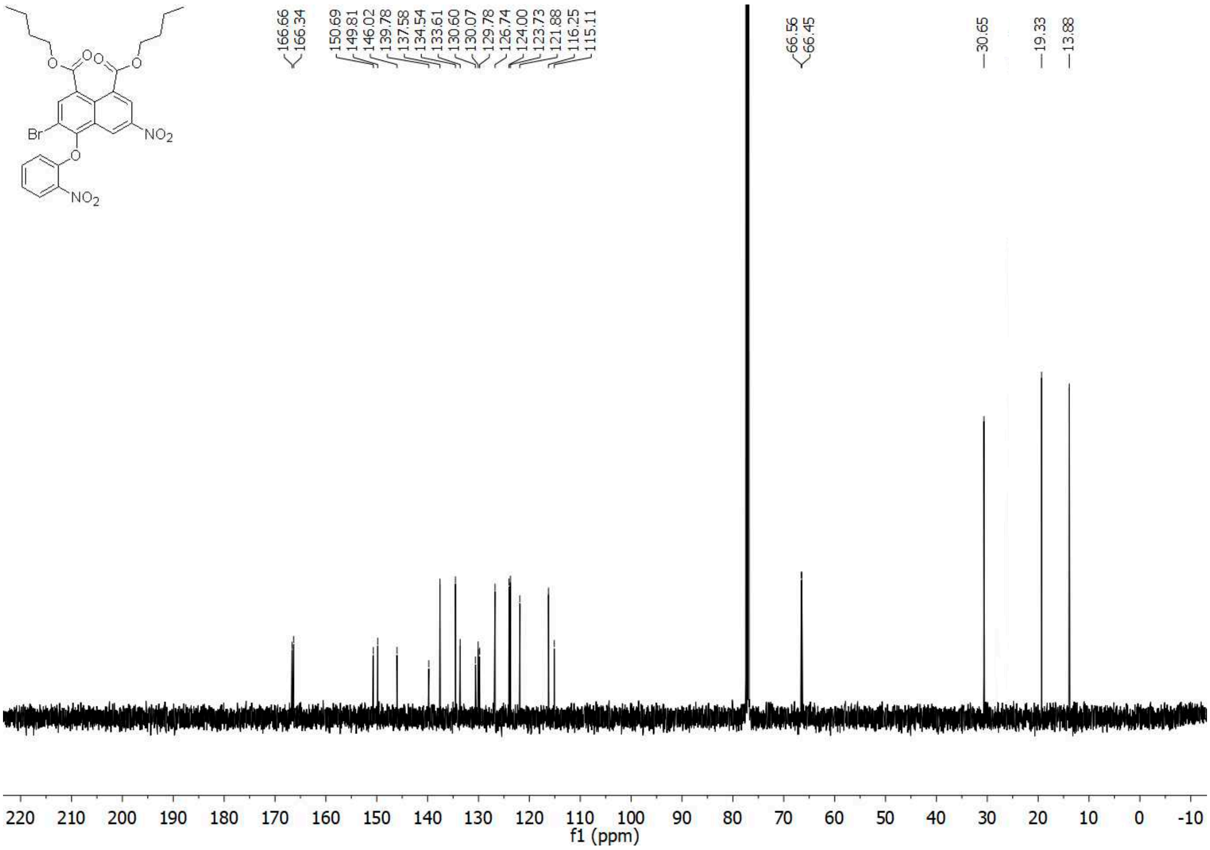

Figure S5: ^{1}H NMR spectrum of the compound **2c** in chloroform-*d*

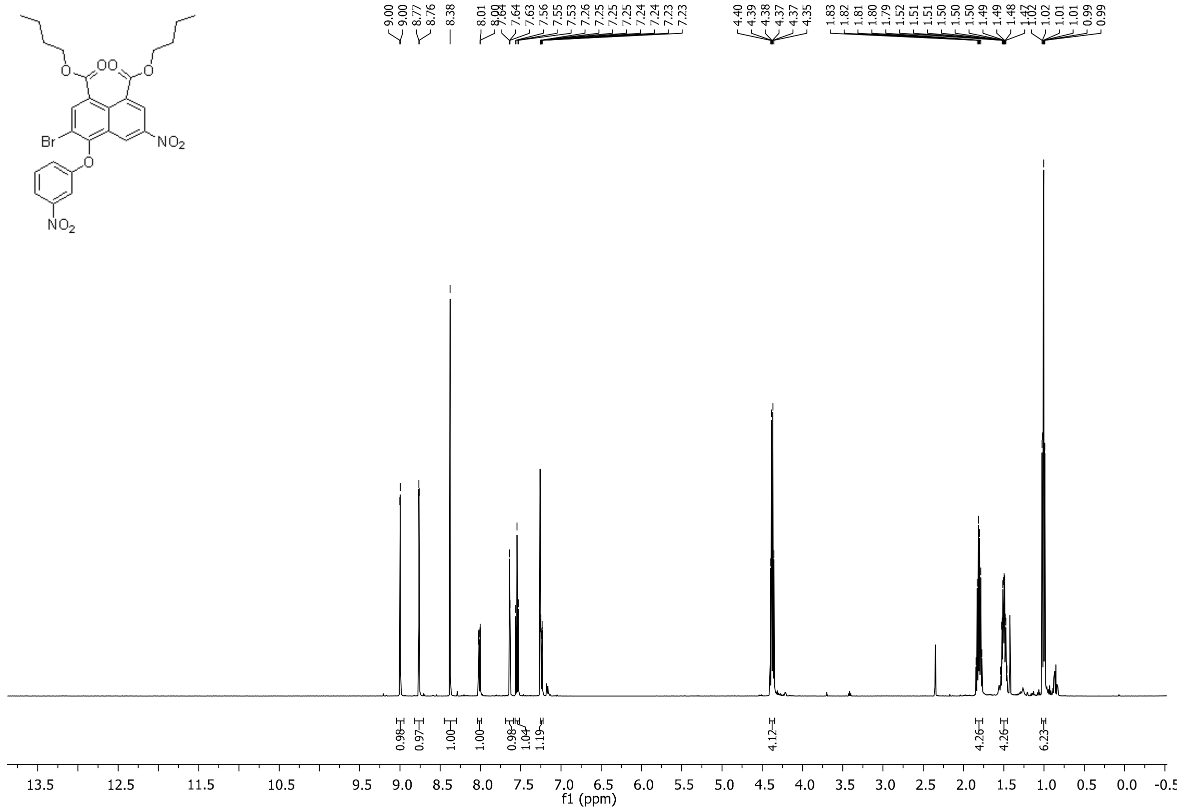

Figure S6: ^{13}C NMR spectrum of the compound **2c** in chloroform-*d*

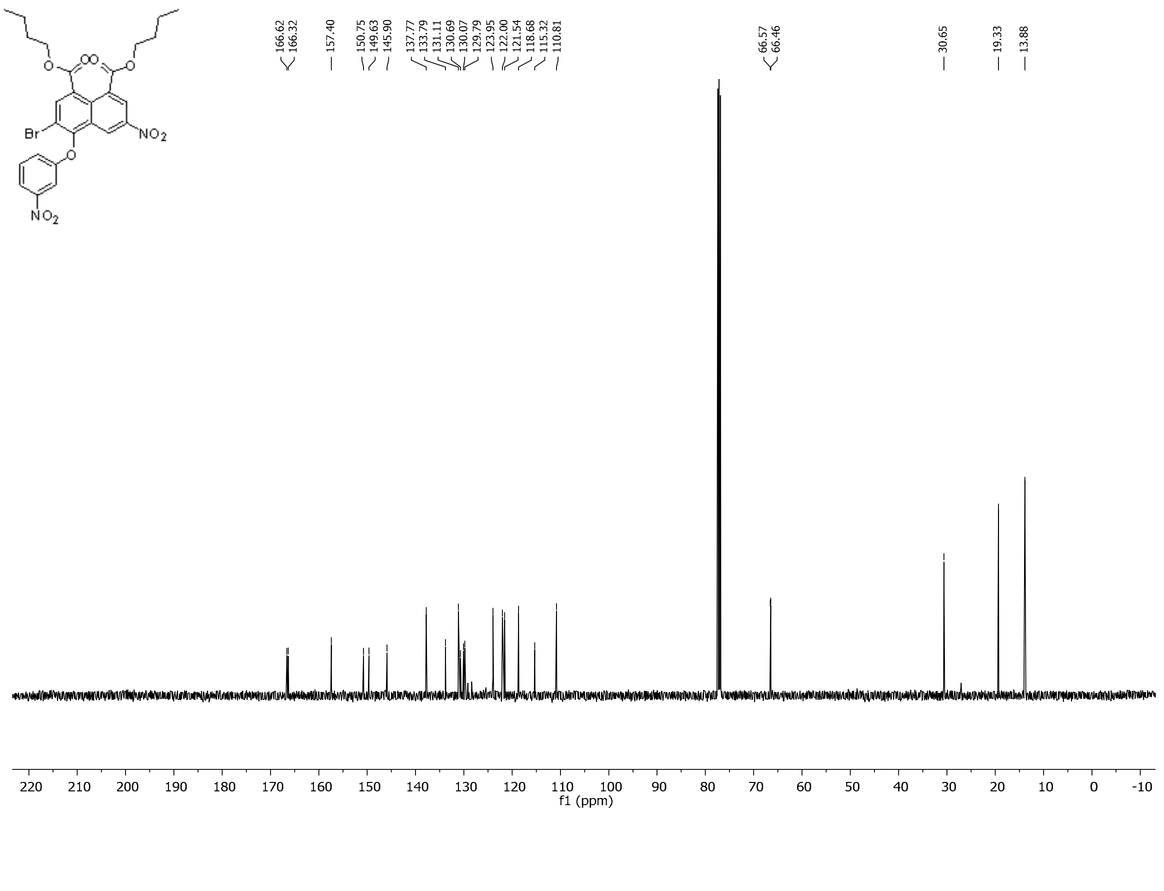

Figure S7: ^{1}H NMR spectrum of the compound **2d** in chloroform-*d*

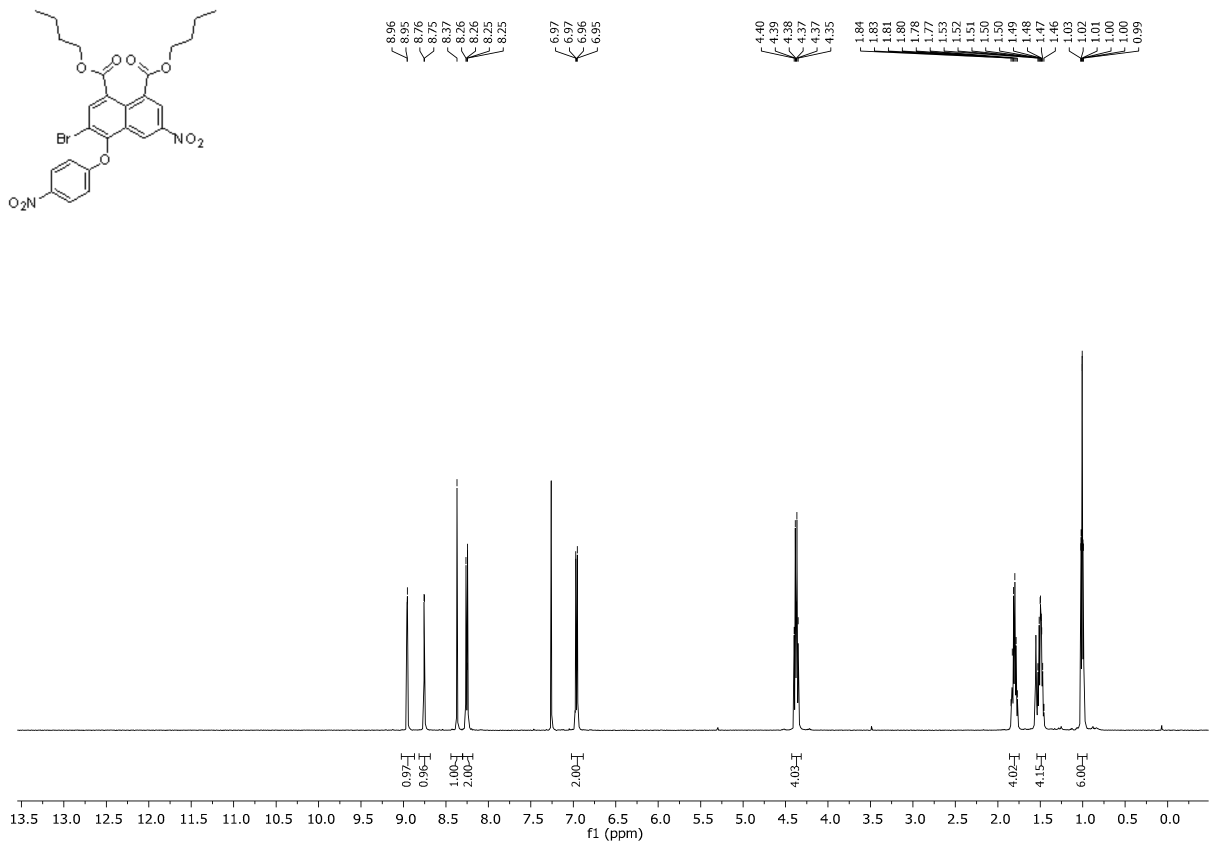

Figure S8: ^{13}C NMR spectrum of the compound **2d** in chloroform-*d*

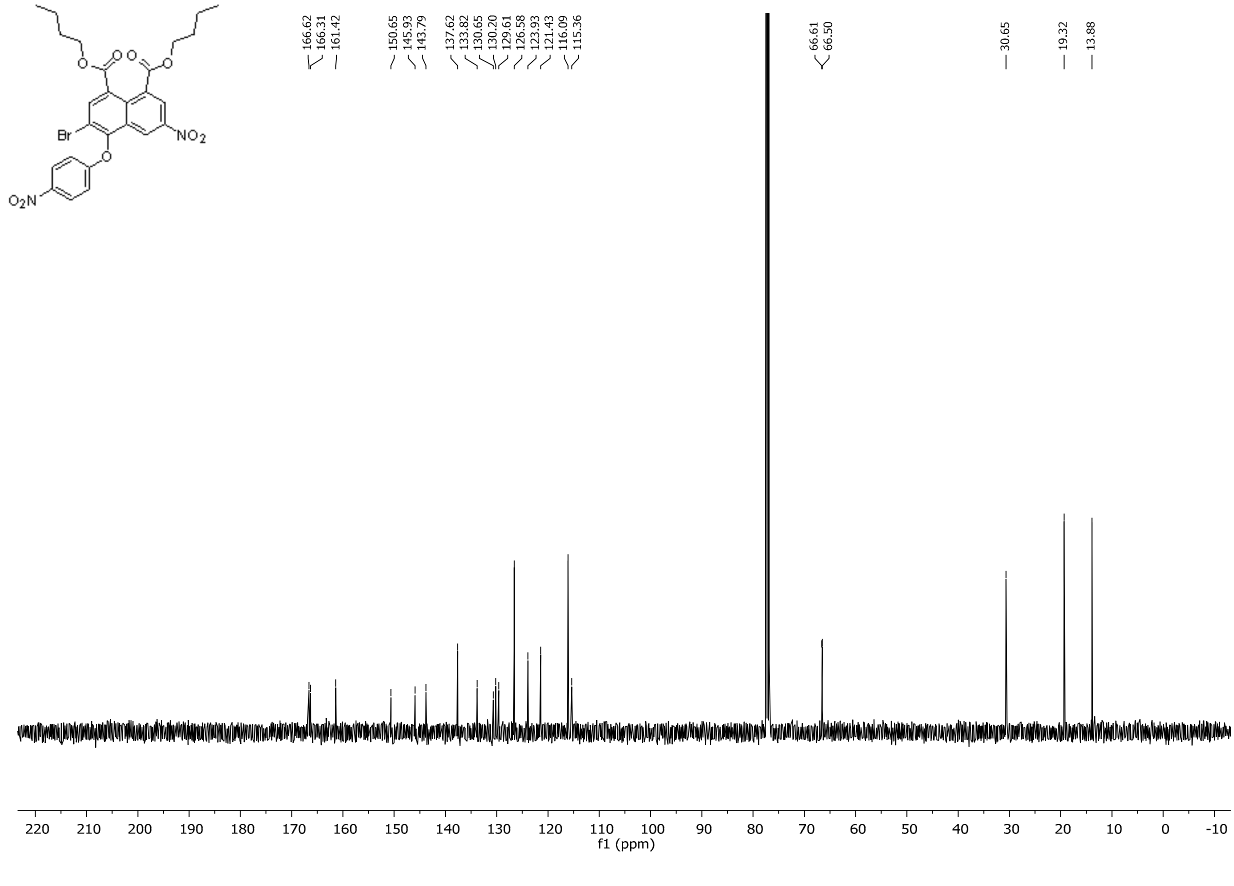

Figure S9: ^{1}H NMR spectrum of the compound **3a** in chloroform-*d*

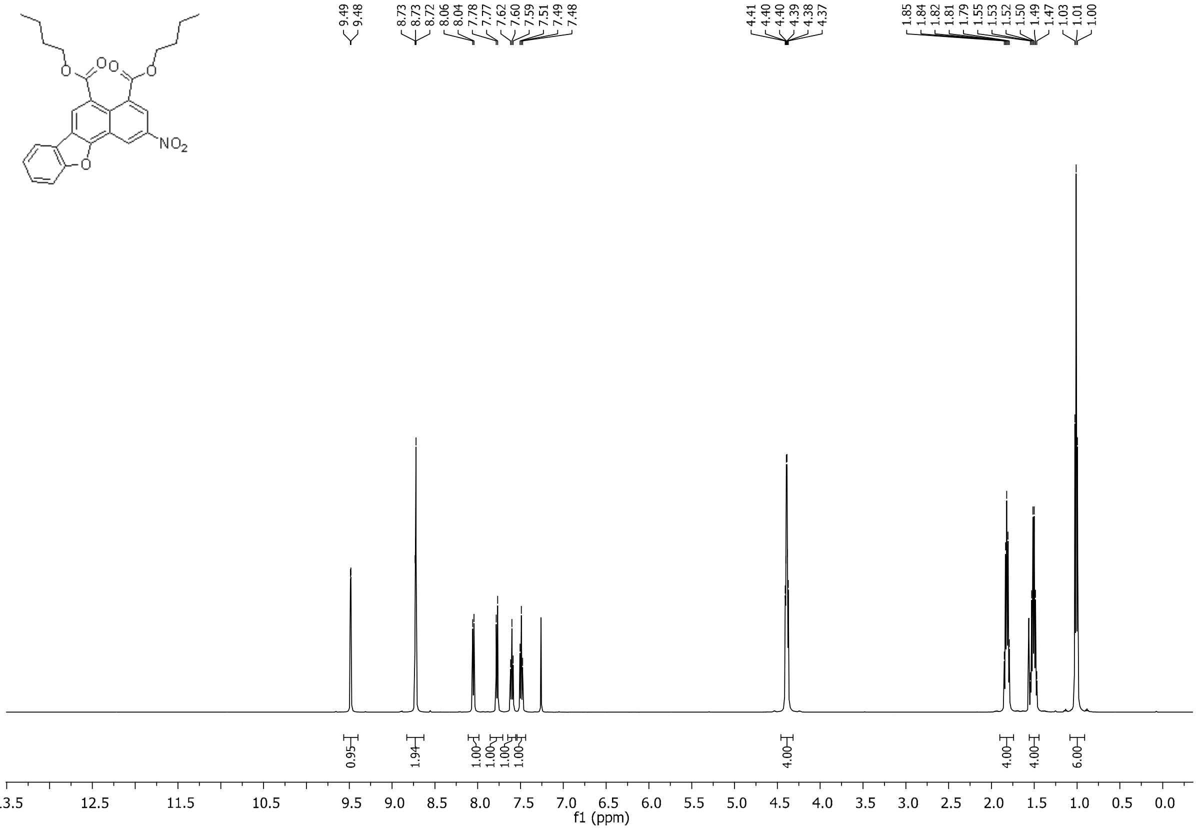

Figure S10: ^{13}C NMR spectrum of the compound **3a** in chloroform-*d*

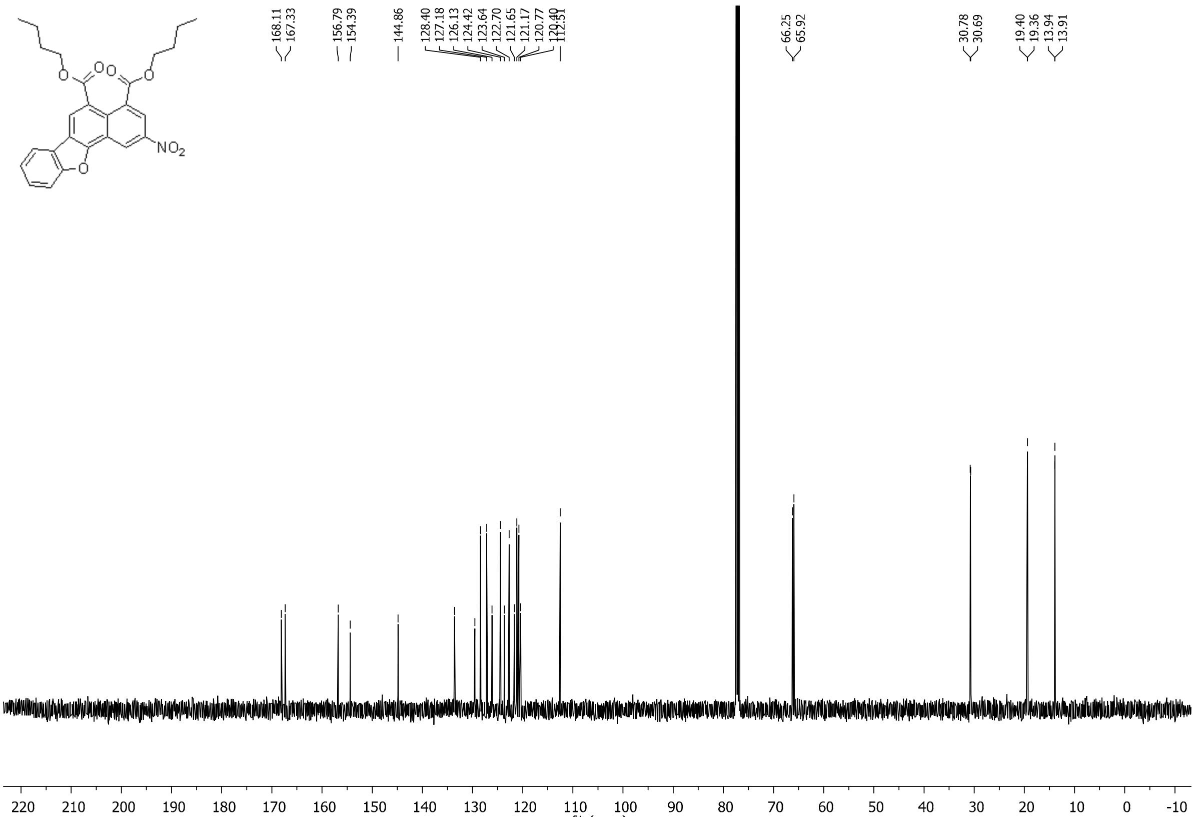

Figure S11: ^{1}H NMR spectrum of the compound **3b** in chloroform-*d*

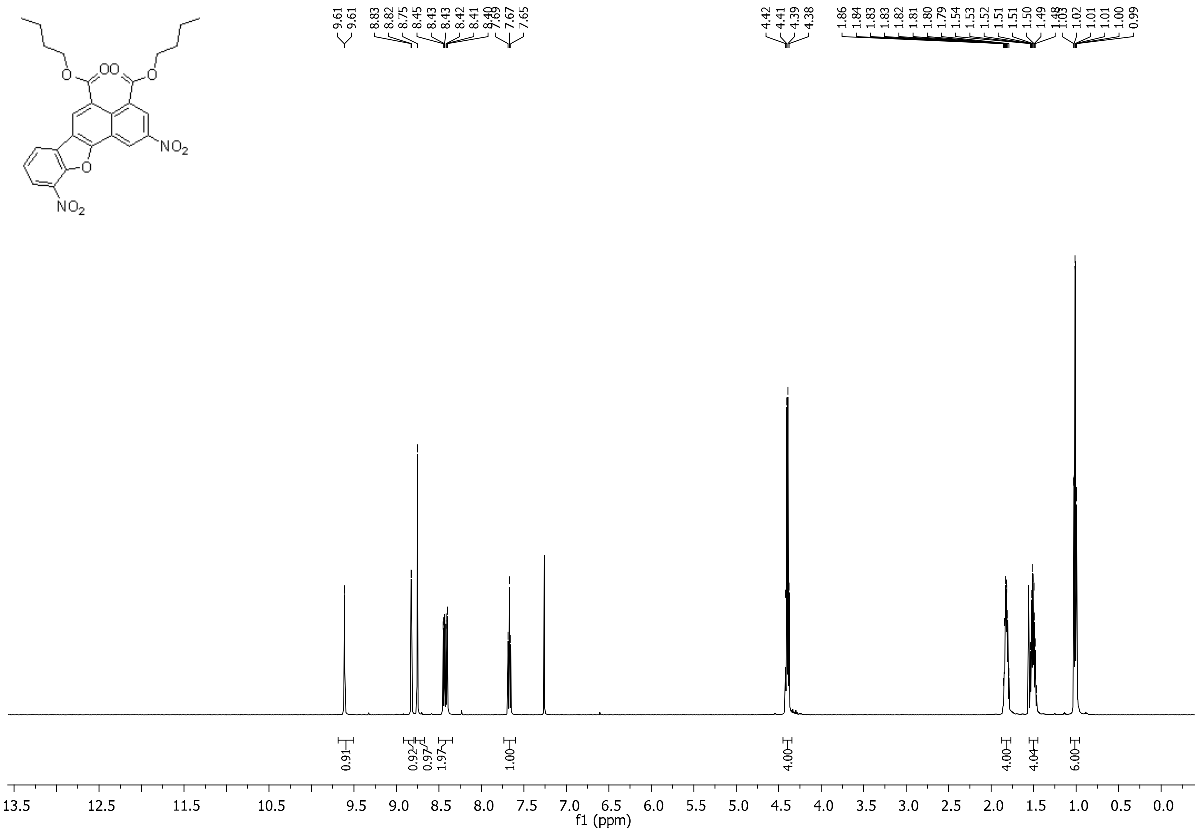

Figure S12: ^{13}C NMR spectrum of the compound **3b** in chloroform-*d*

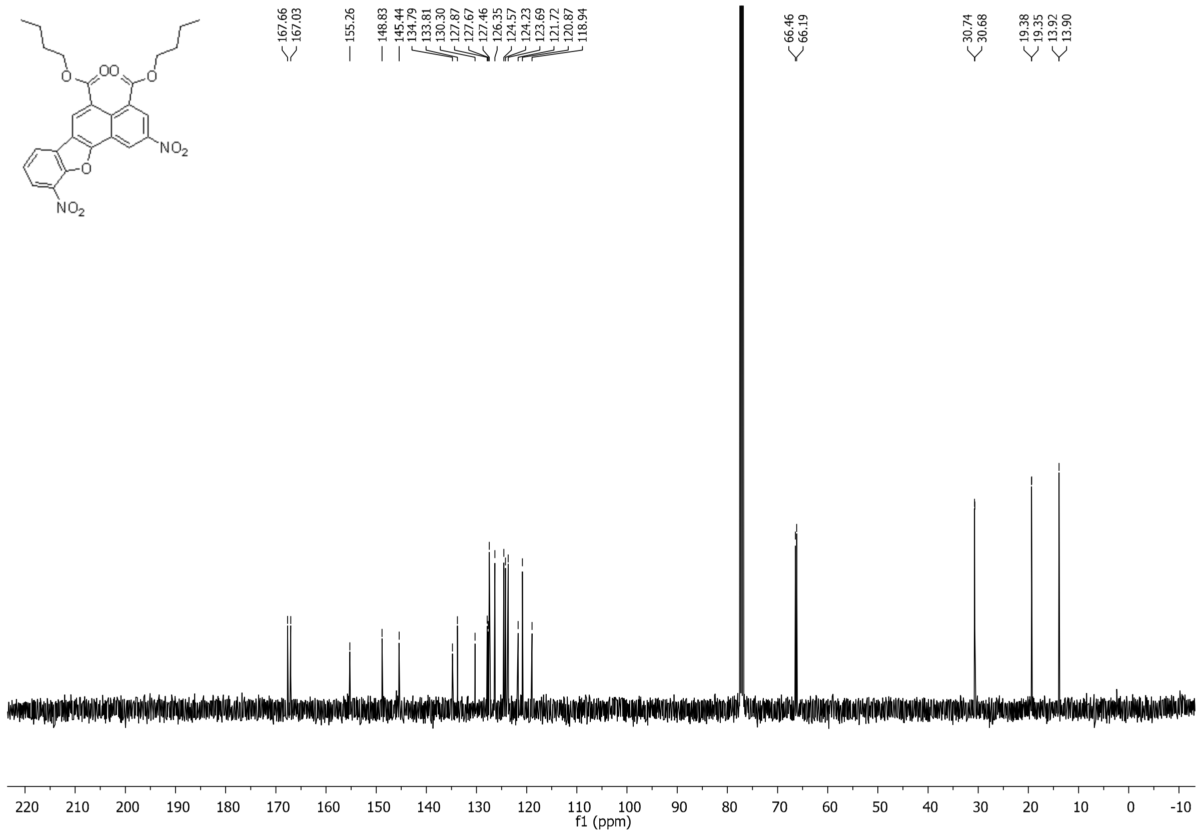

Figure S13: ^{1}H NMR spectrum of the compound **3c** in chloroform-*d*

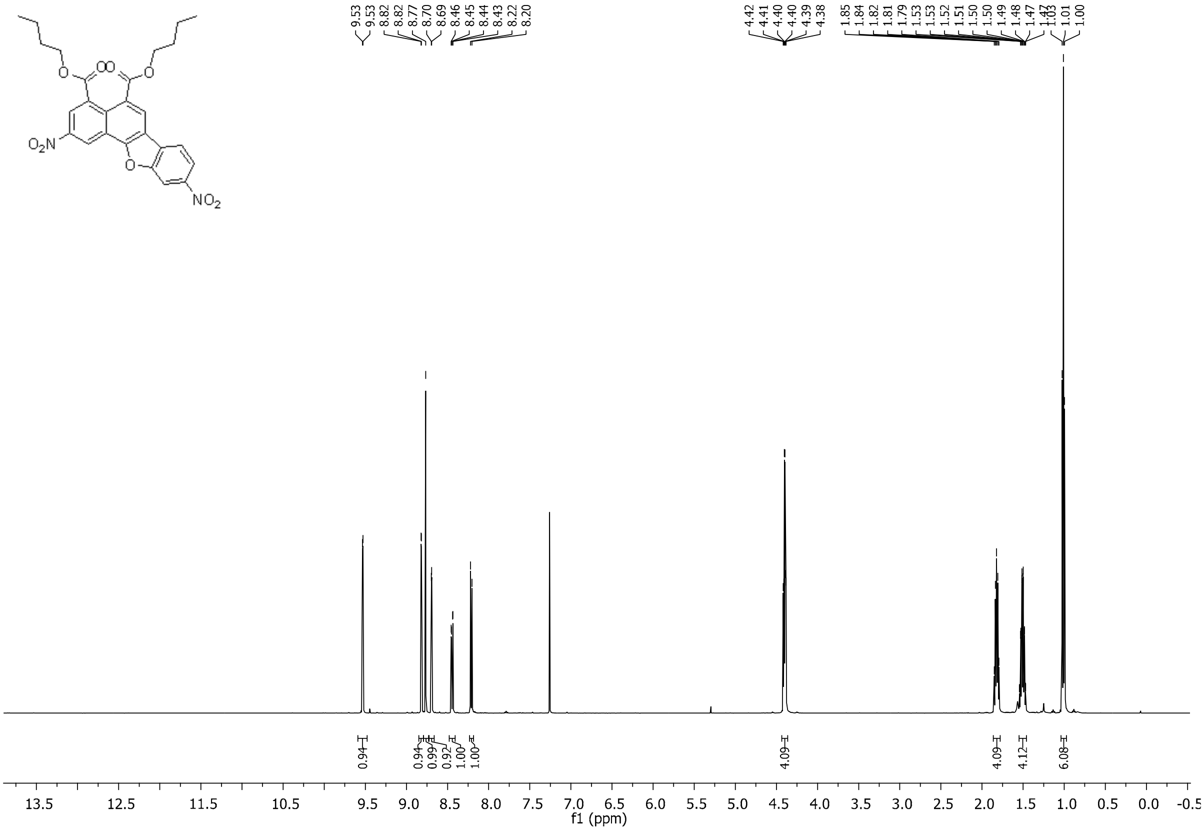

Figure S14: ^{13}C NMR spectrum of the compound **3c** in chloroform-*d*

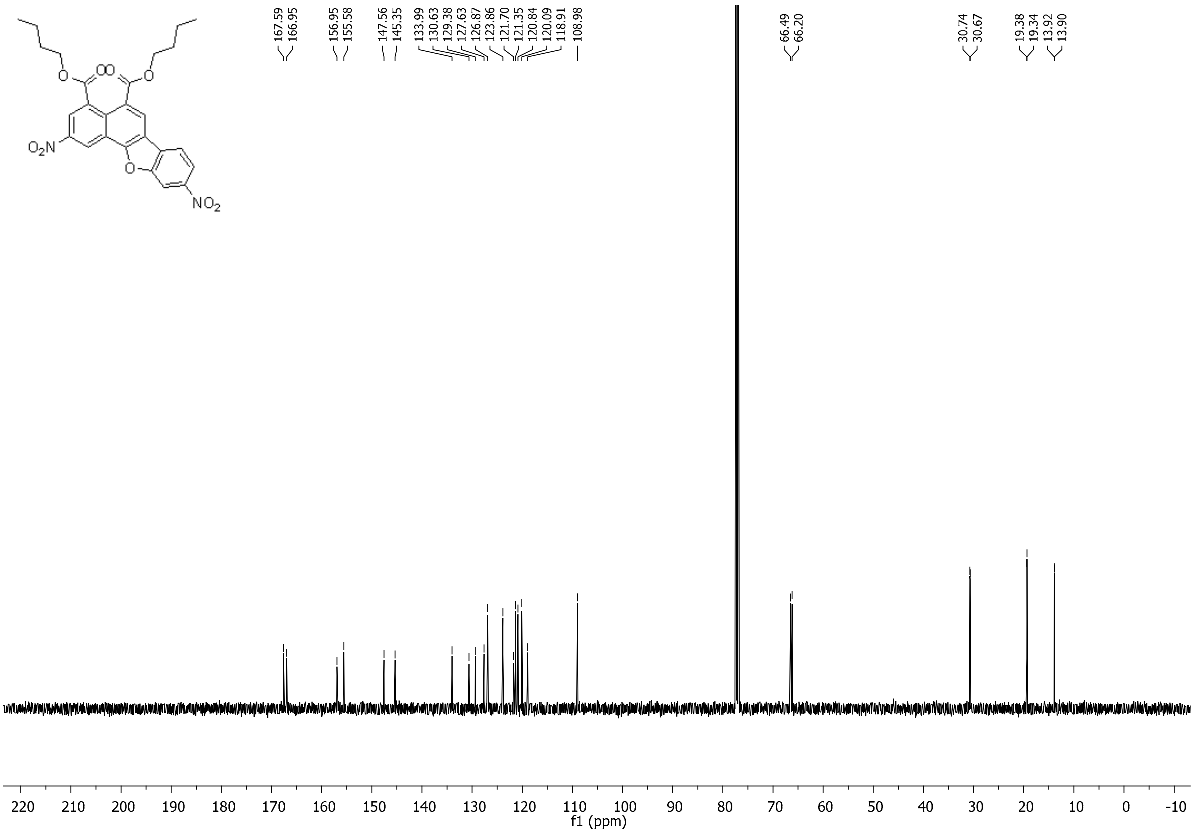

Figure S15: ^{1}H NMR spectrum of the compound **3d** in chloroform-*d*

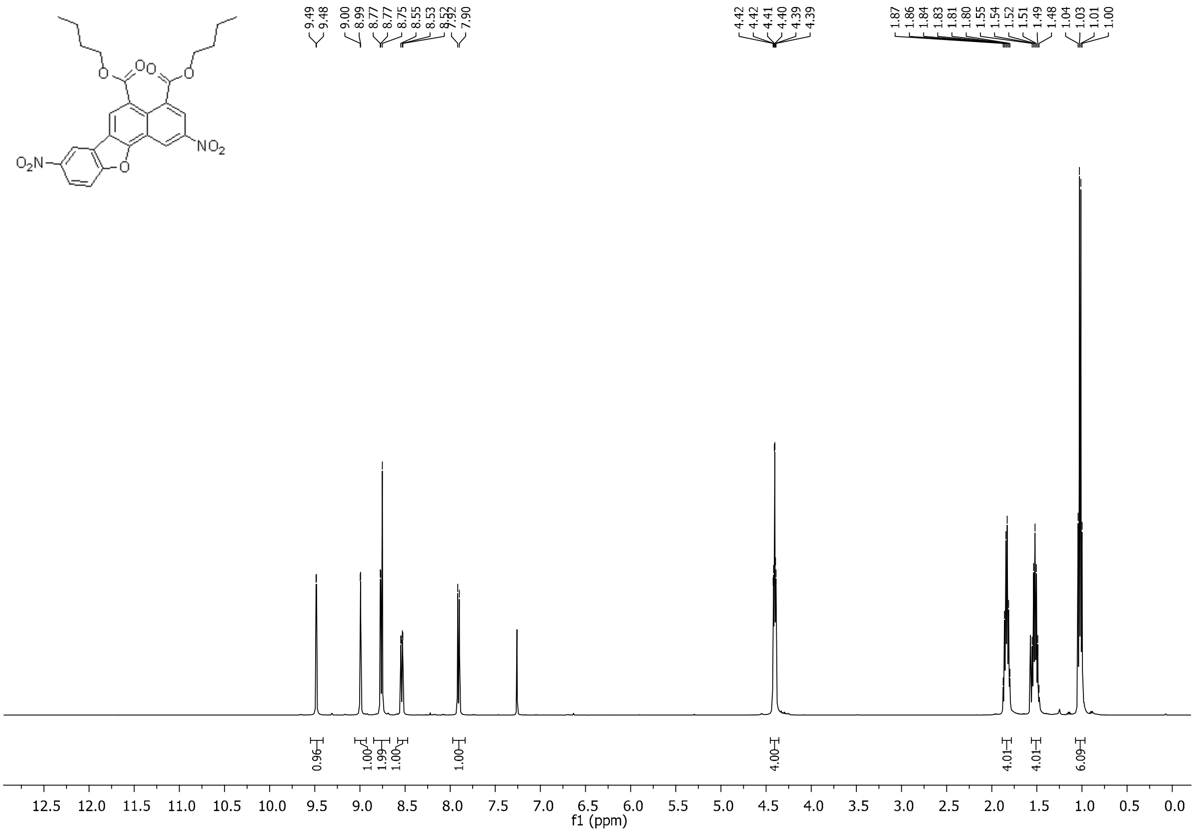

Figure S16: ^{13}C NMR spectrum of the compound **3d** in chloroform-*d*

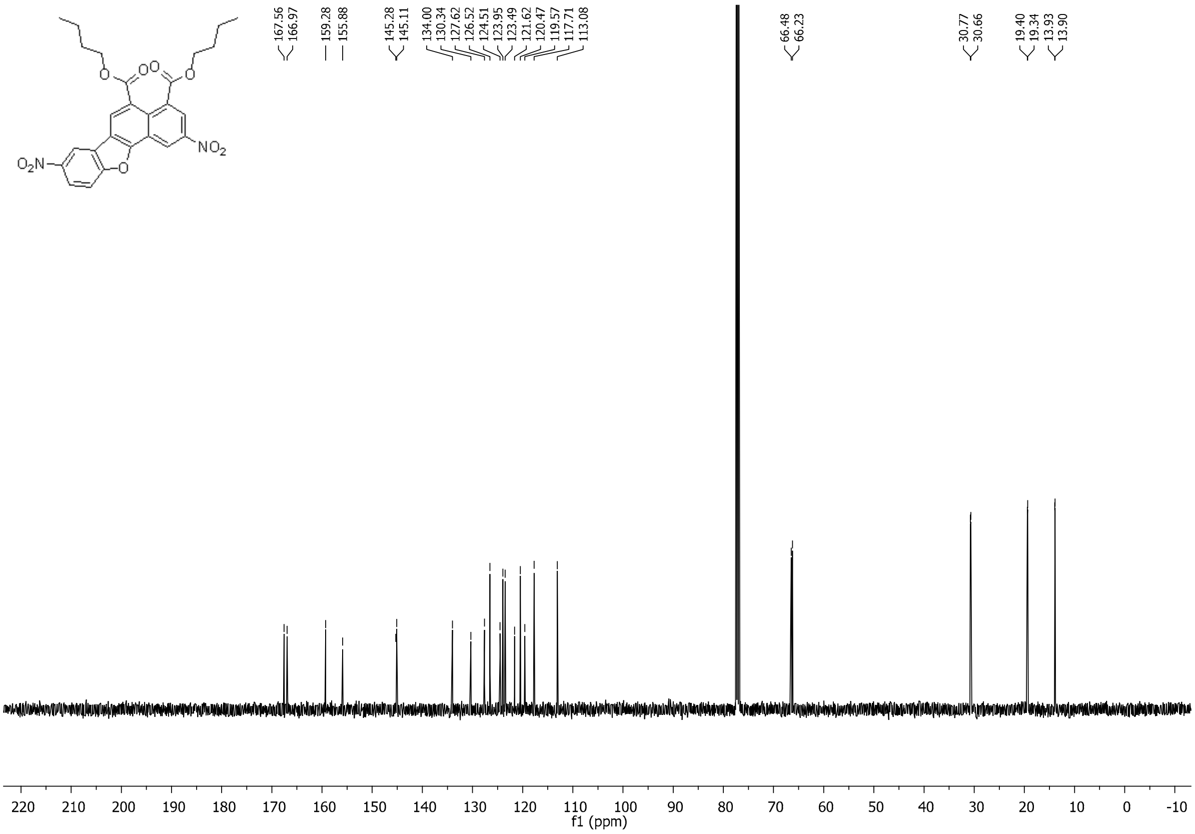

Figure S17: ^{1}H NMR spectrum of the compound **5a** in trifluoroacetic acid-*d*

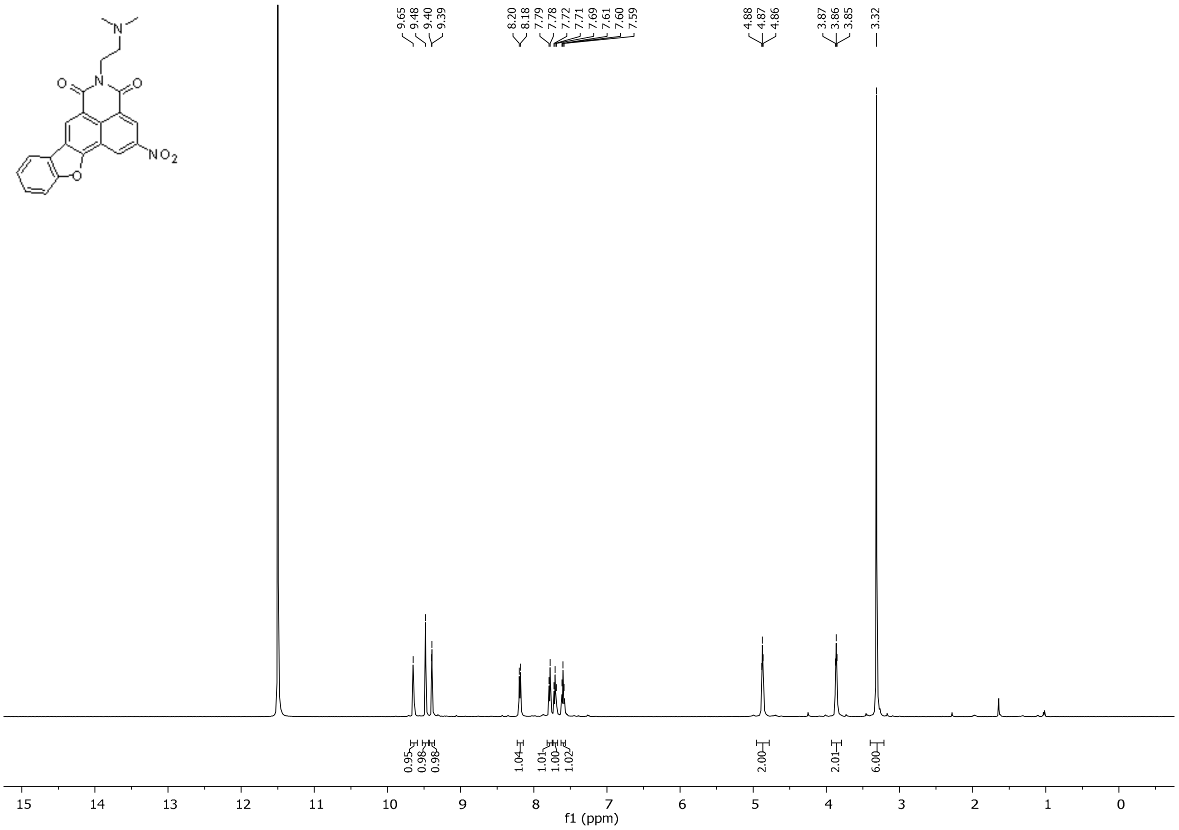

Figure S18: ^{13}C NMR spectrum of the compound **5a** in trifluoroacetic acid-*d*

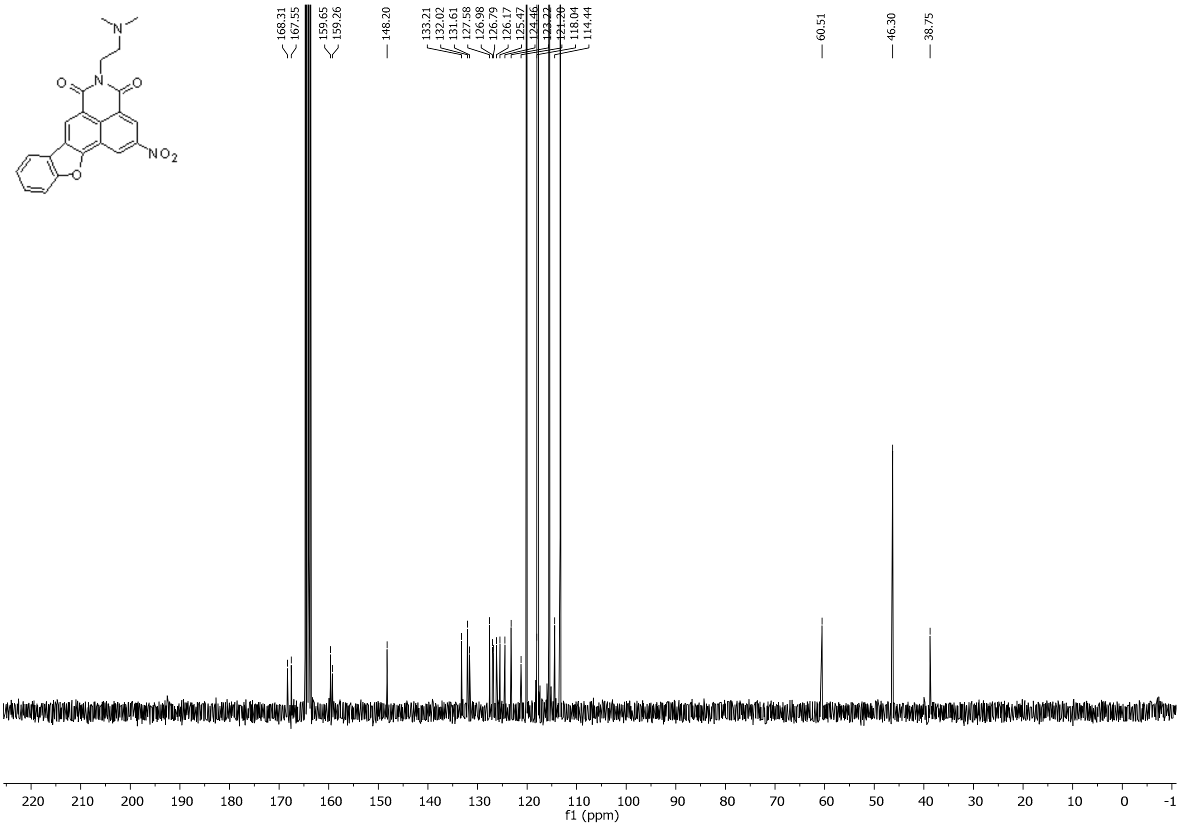

Figure S19: ^{1}H NMR spectrum of the compound **5b** in trifluoroacetic acid-*d*

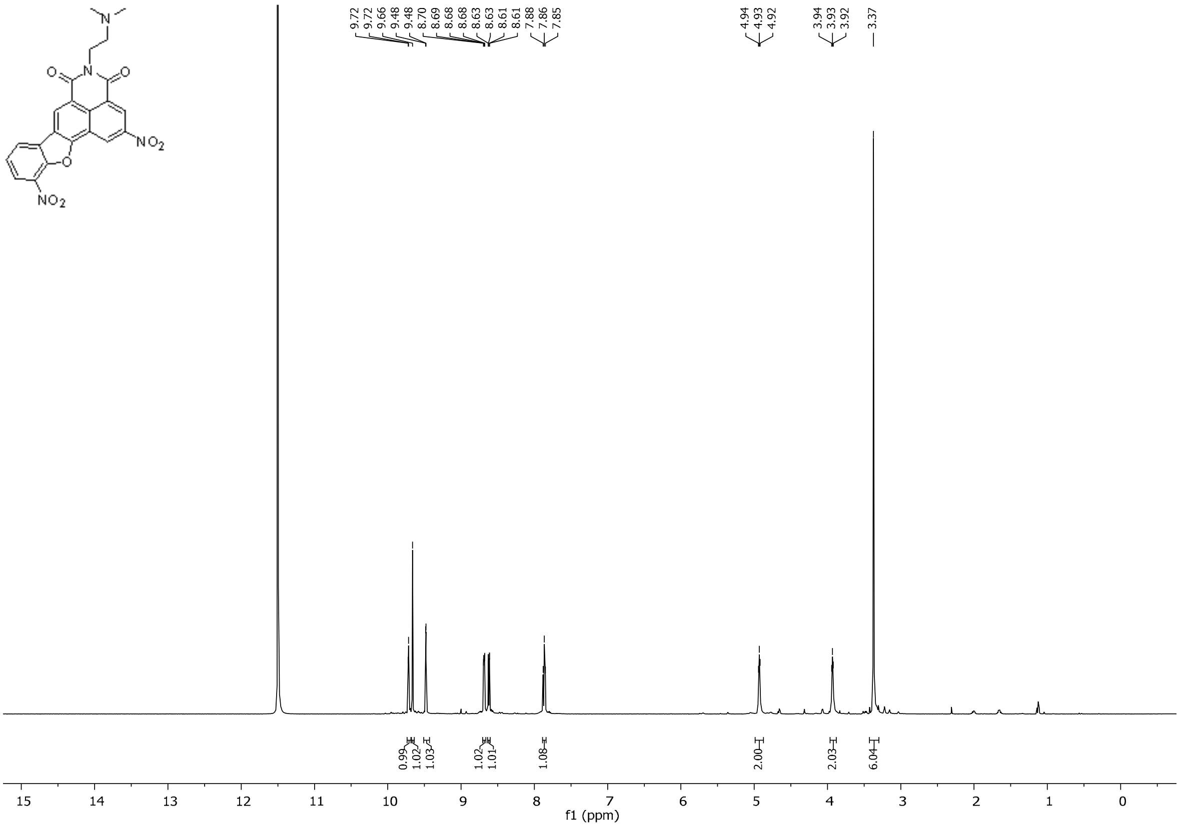

Figure S20: ^{13}C NMR spectrum of the compound **5b** in trifluoroacetic acid-*d*

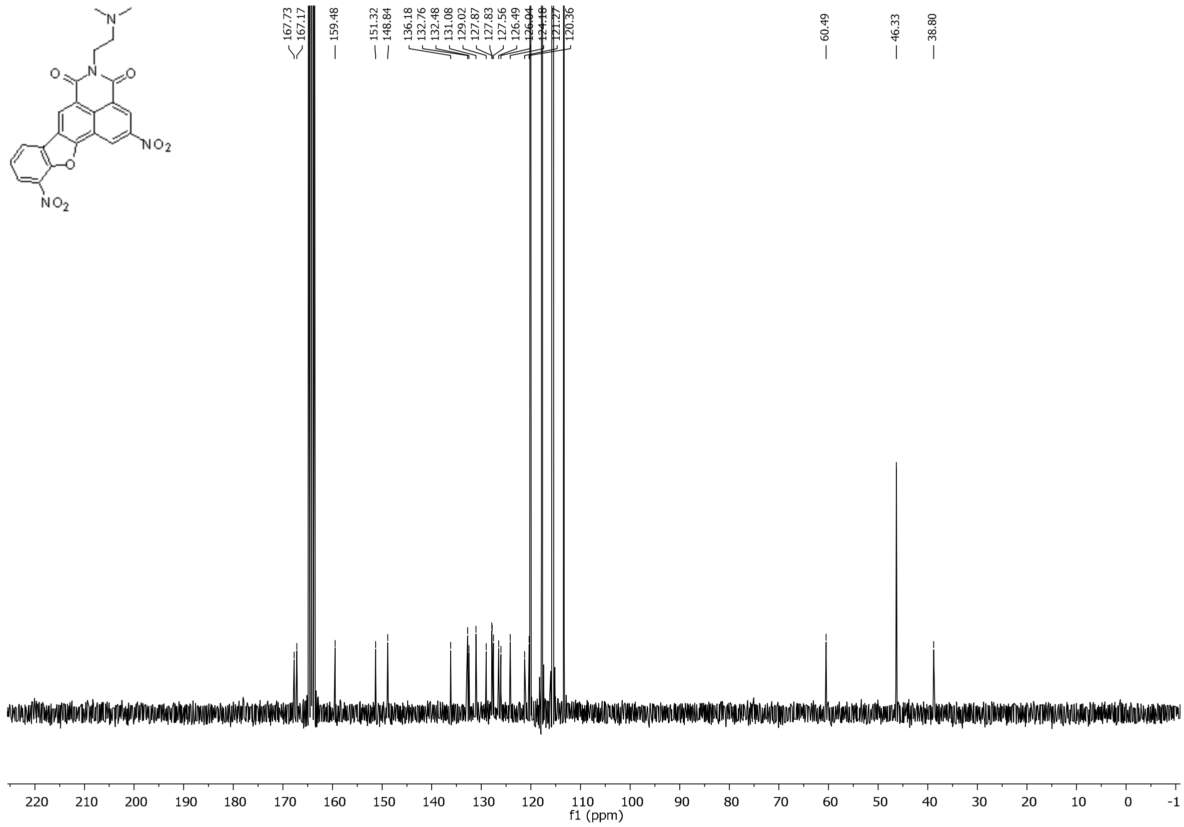

Figure S21: ^{1}H NMR spectrum of the compound **5c** in trifluoroacetic acid-*d*

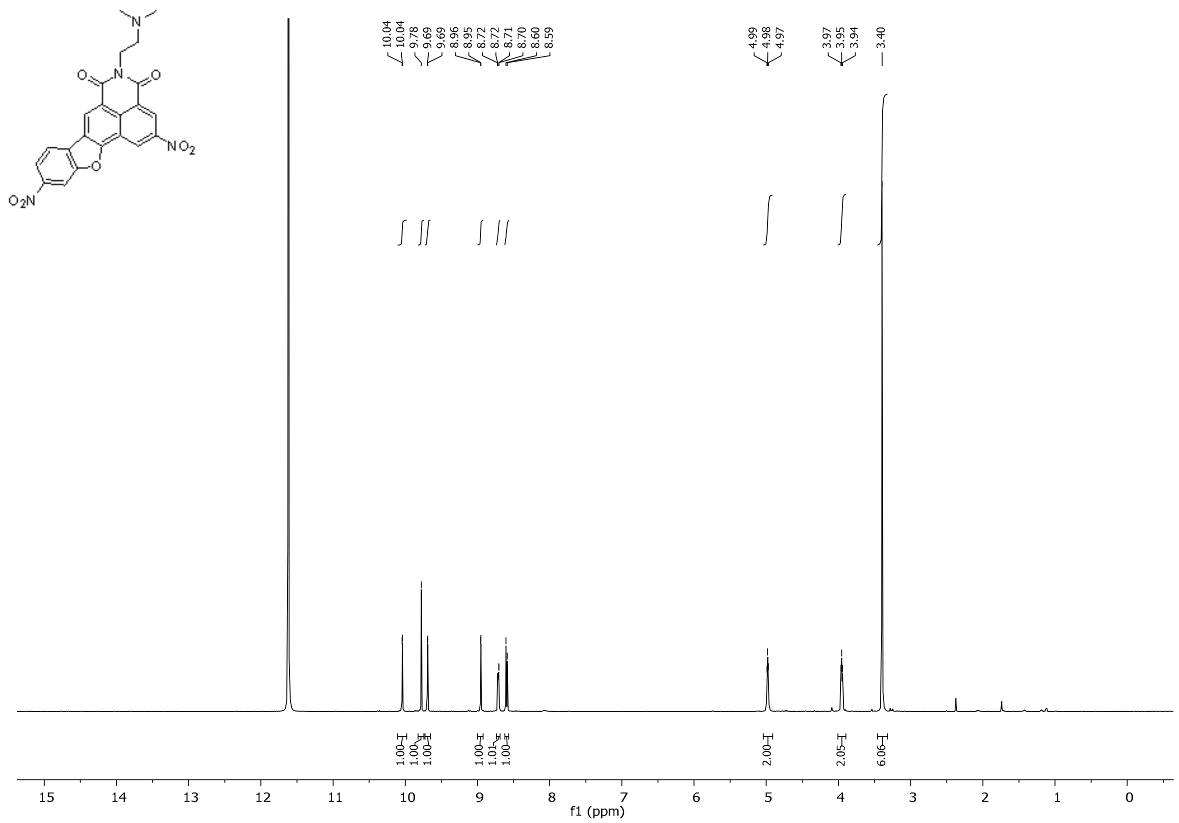

Figure S22: ^{13}C NMR spectrum of the compound **5c** in trifluoroacetic acid-*d*

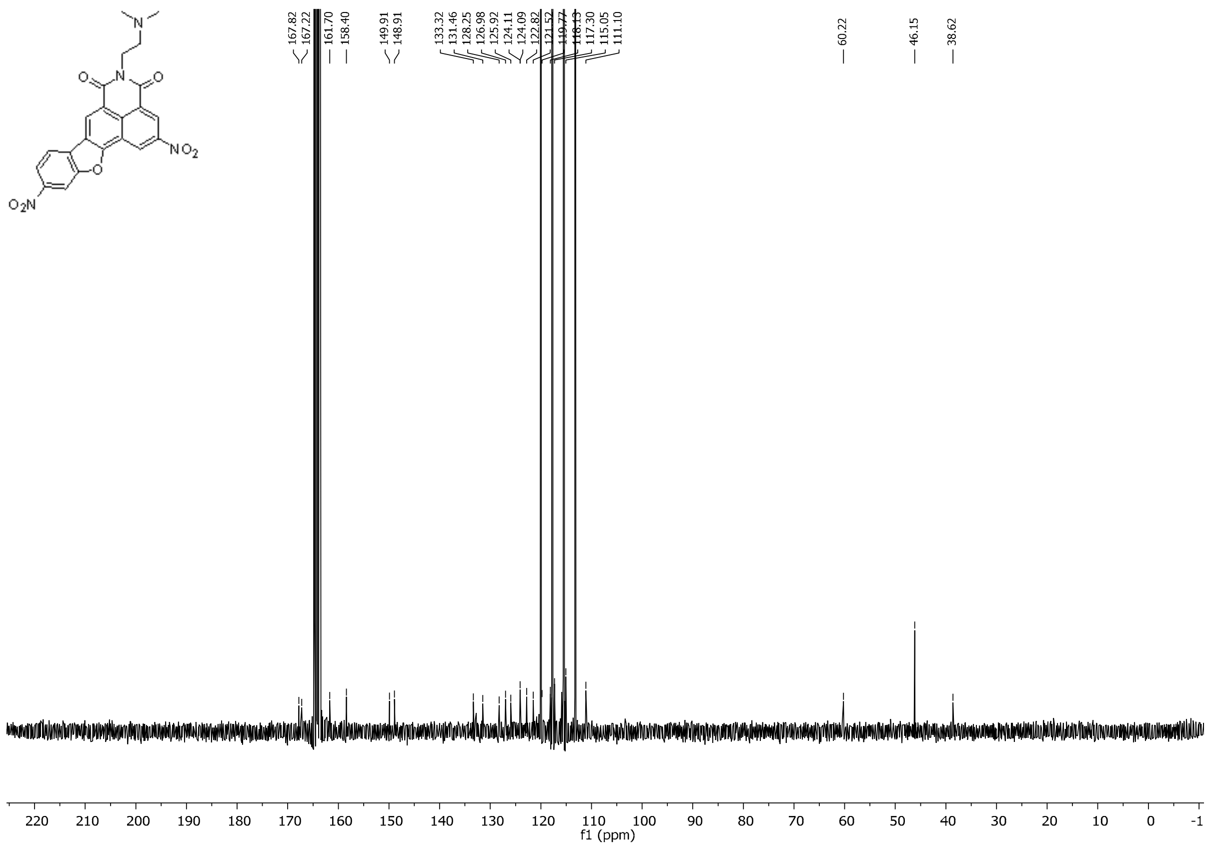

Figure S23: ^{1}H NMR spectrum of the compound **5d** in trifluoroacetic acid-*d*

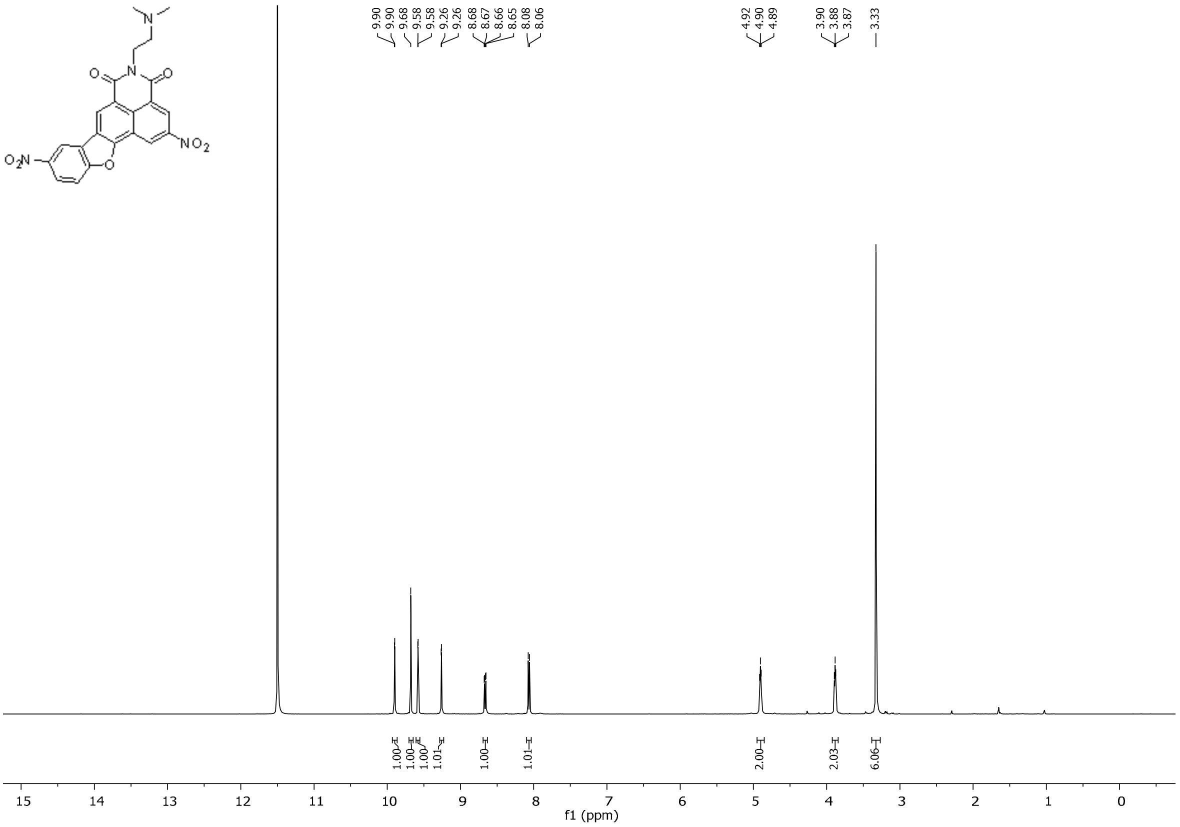

Figure S24: ^{13}C NMR spectrum of the compound **5d** in trifluoroacetic acid-*d*

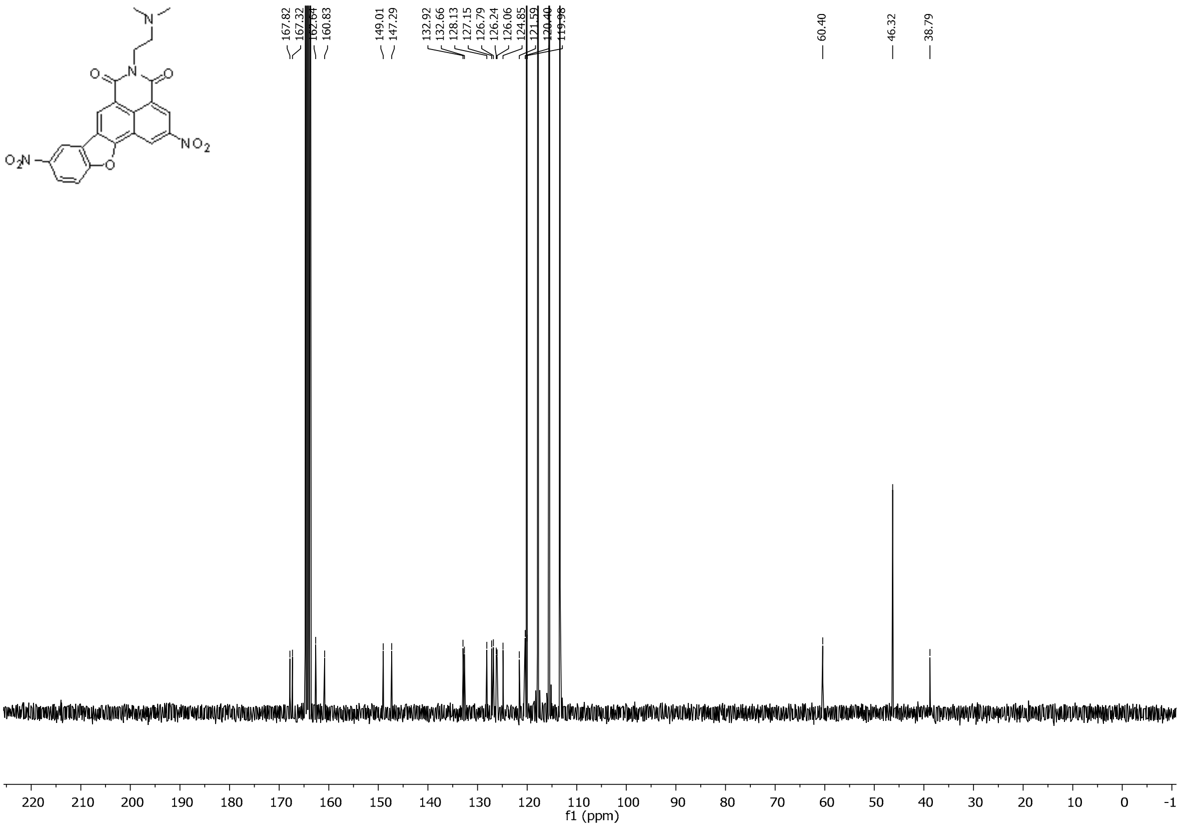

Figure S25: HRMS spectrum of compound **5a**

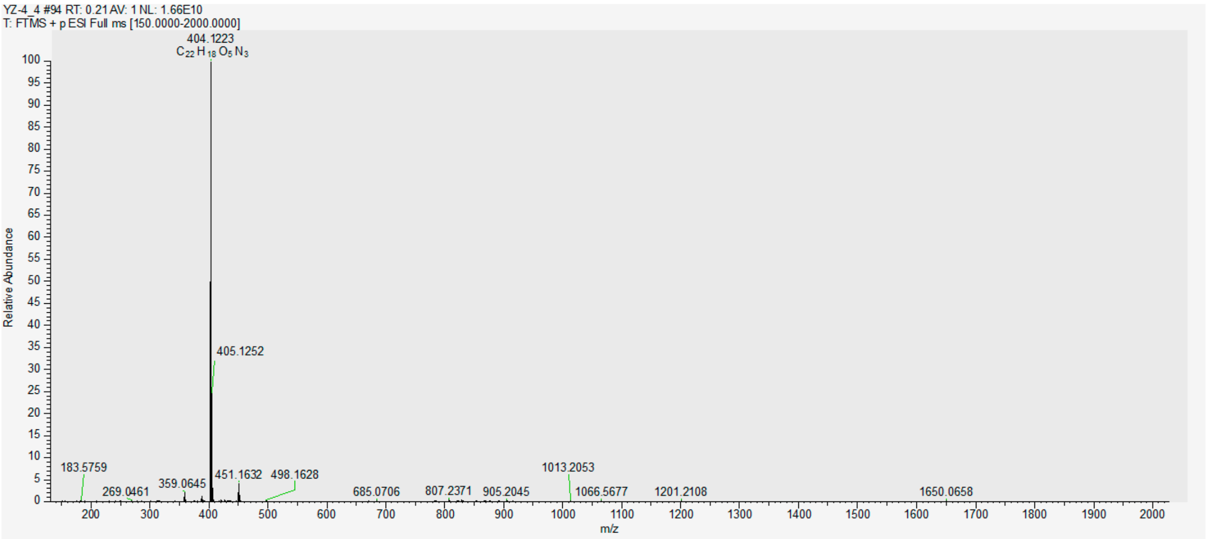

Figure S26: HRMS spectrum of compound **5b**

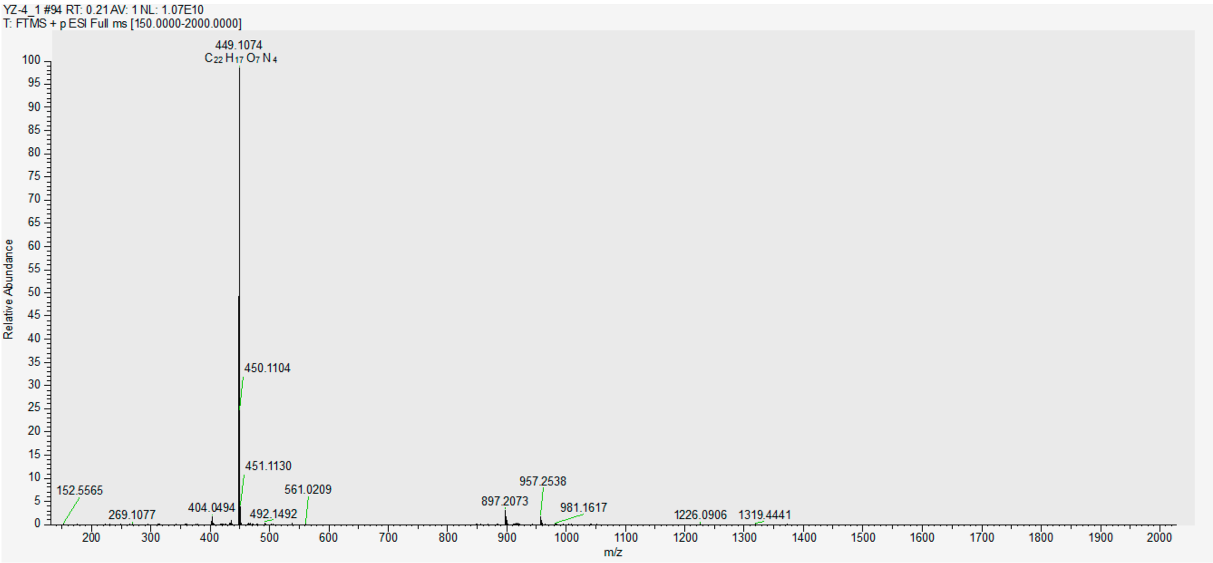

Figure S27: HRMS spectrum of compound **5c**

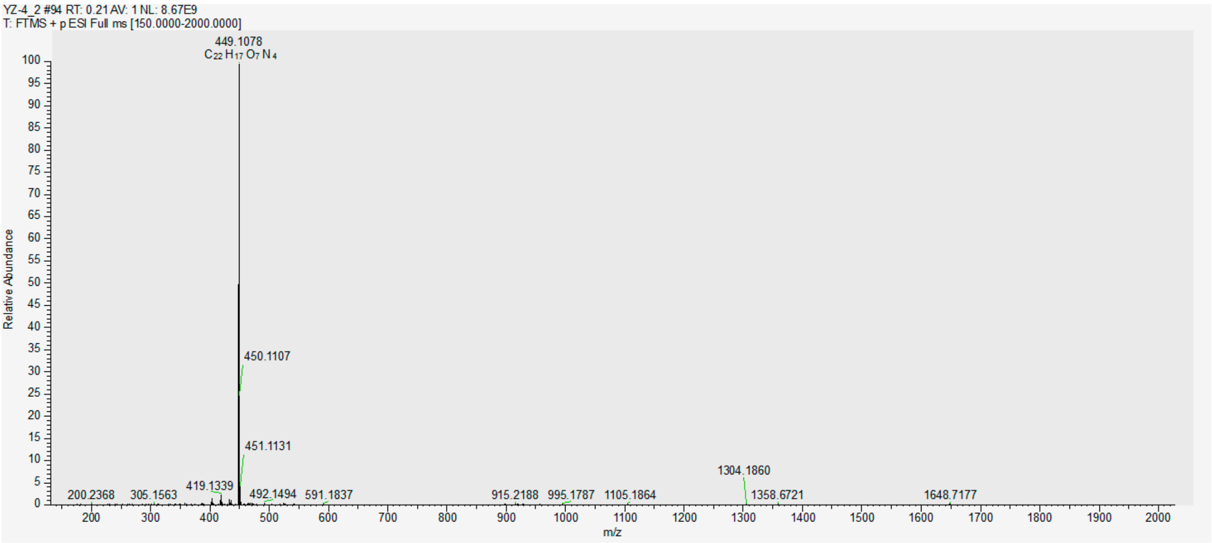

Figure S28: HRMS spectrum of compound **5d**

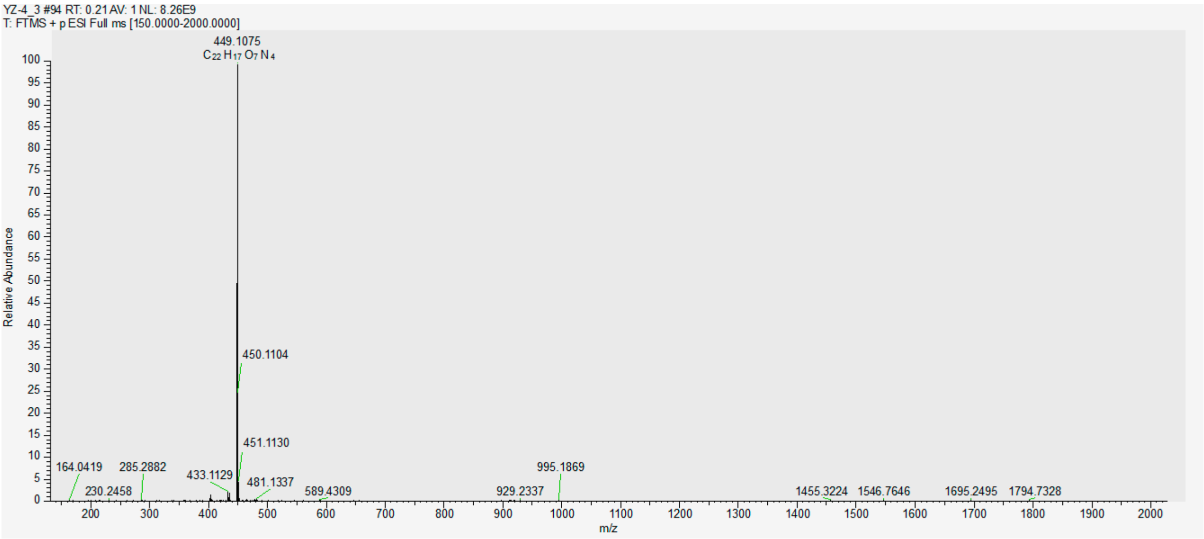

Figure S29: Dose–response curves of A549, H1299, and MRC-5 cells following 72 h exposure to different concentrations of compounds **5a** (a), **5b** (b), **5c** (c), and **5d** (d).

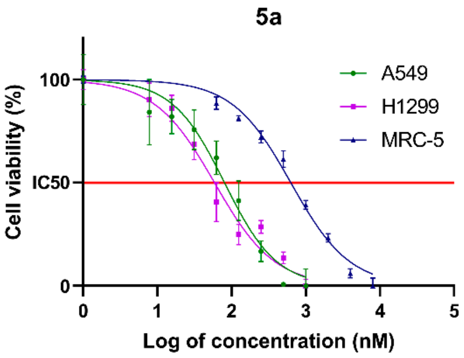

(**a**)

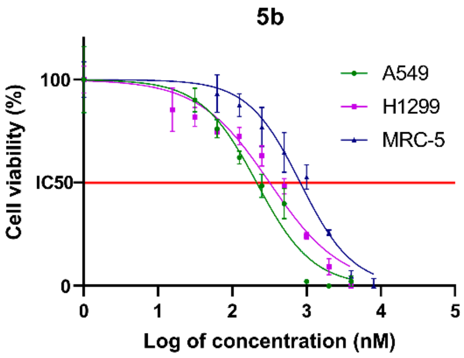

(**b**)

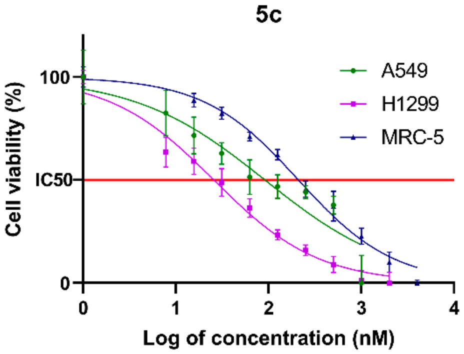

(**c**)

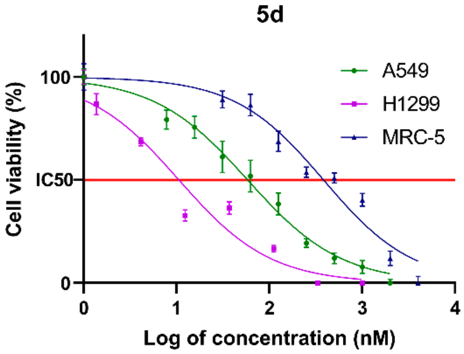

(**d**)

Figure S30: Magnified views of representative cells from immunofluorescence analysis showing γH2AX foci (red), EdU incorporation (green), and DAPI-stained nuclei (blue) after 24 and 48 h treatment with **5d** at IC_{50} and IC_{75} concentrations in A549 (a) and H1299 (b) cells.

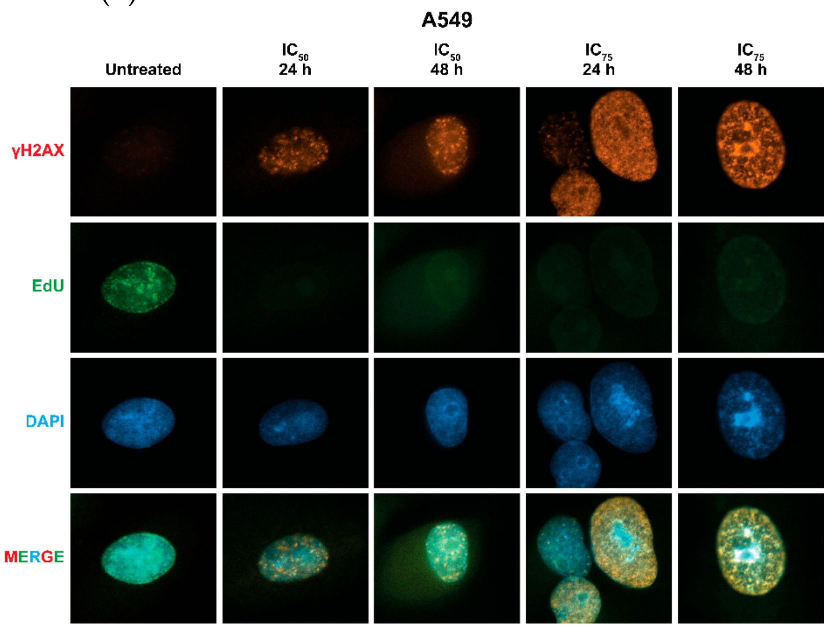

(**a**)

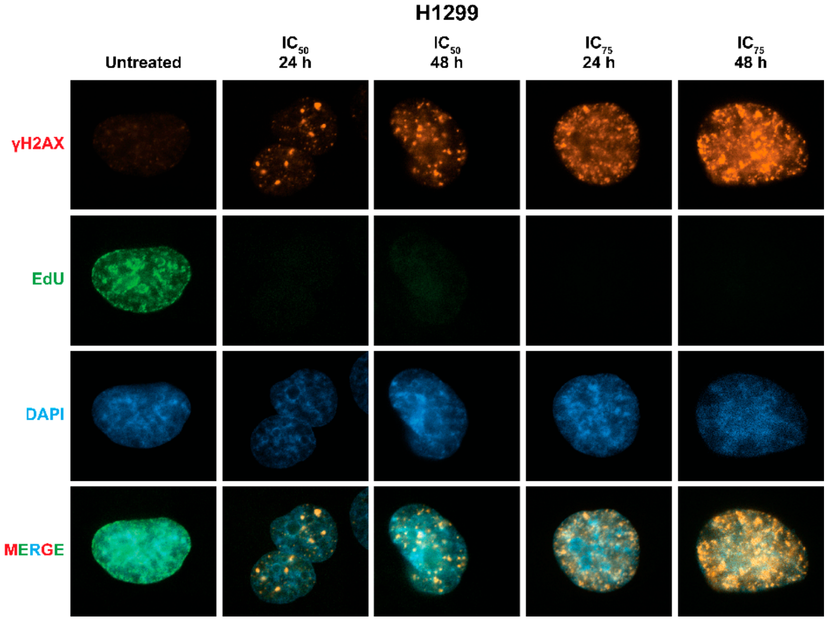

(b)

Figure S31: Magnified views of representative cells showing LC3 puncta formation (green) and DAPI-stained nuclei (blue) after 24 h treatment with **5d** at IC_{50} and IC_{75} concentrations in A549 (a) and H1299 (b) cells.

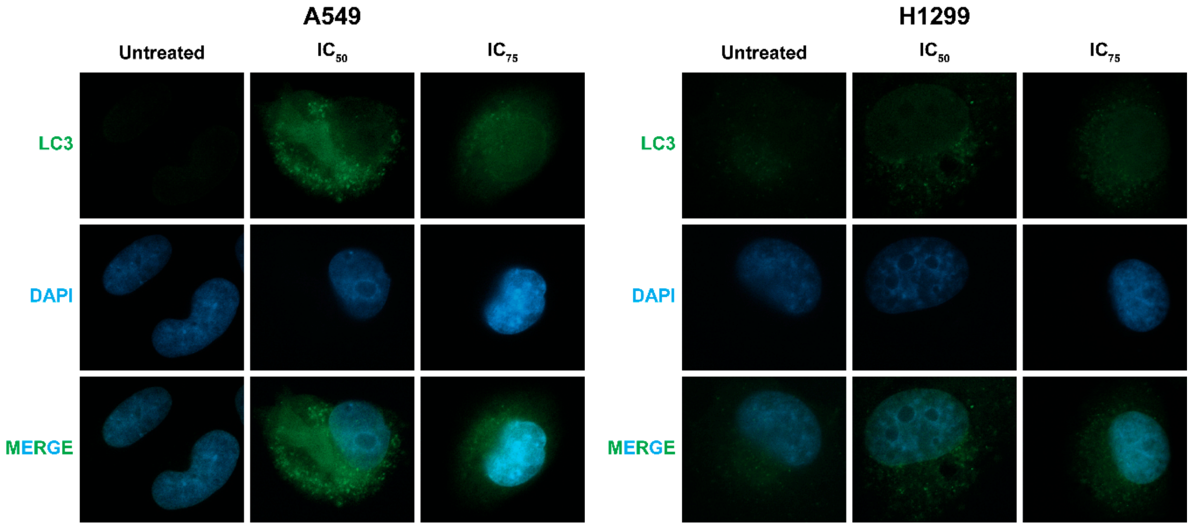

(a) (b)
